# Deubiquitinase USP7 stabilizes the histone demethylase KDM5B and promotes the progression of renal fibrosis through the TSC1/mTOR axis

**DOI:** 10.1186/s43556-026-00531-3

**Published:** 2026-08-05

**Authors:** Shiqi Lv, Ziyan Shen, Yulu Gu, Yulin Wang, Jiayi Wang, Han Zhang, Hong Liu, Jing Chen, Cheng Zhu, Xinhui Huang, Tongqiang Liu, Xiaoqiang Ding, Xiaoyan Zhang

**Affiliations:** 1 Department of Nephrology, Zhongshan Hospital, Fudan University, No. 180 Fenglin Road, Shanghai, 200032 China; 2 Shanghai Medical Center of Kidney Disease, 179 Fenglin Road, Shanghai, 200032 China; 3 Shanghai Key Laboratory of Kidney and Blood Purification, 179 Fenglin Road, Shanghai, 200032 China; 4 Shanghai Institute of Kidney and Dialysis, 179 Fenglin Road, Shanghai, 200032 China; 5 Department of Nephrology, The Second People’s Hospital of Changzhou, the Third Affiliated Hospital of Nanjing Medical University, 68 Gehu Middle Road, Changzhou, Jiangsu 213001 China

**Keywords:** Ubiquitin-specific protease 7 (USP7), Lysine-specific demethylase 5B (KDM5B), Histone methylation, Kidney fibrosis, Deubiquitination

## Abstract

Ubiquitin-specific protease 7 (USP7) is a deubiquitinase that plays critical regulatory roles in multiple signaling pathways by preventing the ubiquitin-mediated degradation of its substrates. Dysregulated expression of USP7 is implicated in tumor progression; however, its role in renal fibrosis remains unclear. In the present study, USP7 was observed to be significantly upregulated in the kidneys of patients with chronic kidney disease (CKD), which correlated with fibrotic lesions and renal dysfunction. Both genetic depletion and pharmacological blockade of USP7 significantly attenuated fibroblast activation and extracellular matrix deposition in two mouse models of kidney fibrosis—unilateral ureteral obstruction and unilateral renal ischemia–reperfusion injury models, indicating a pro-fibrotic function of USP7. Mechanistically, intergrated proteomic sequencing and phosphoproteomic sequencing revealed that USP7 modulated the tuberous sclerosis complex 1 (TSC1)-mTOR pathway. USP7 knockdown restored TSC1 expression and inhibited mTOR activation. However, USP7 did not directly interact with TSC1; instead, it deubiquitinated and stabilized lysine-specific demethylase 5B (KDM5B), which subsequently reduced histone H3K4me3 modification at the *Tsc1* promoter to repress its transcription. Conversely, the inhibition of USP7 promoted KDM5B degradation, thereby restoring TSC1 expression and suppressing mTOR-driven fibrogenesis. Thus, these findings identify USP7 as a critical promoter of renal fibrosis, acting at least in part through the KDM5B-TSC1-mTOR axis. This highlights USP7 as a potential therapeutic target for CKD.

**Supplementary Information:**

The online version contains supplementary material available at 10.1186/s43556-026-00531-3.

## Introduction

Chronic kidney disease (CKD) affects approximately 14.2% of the global population, with a continuous increase in its incidence annually [1]. Renal interstitial fibrosis is a critical pathological process contributing to CKD progression [2]. Unlike fibrosis in other organs such as the lung and liver, the complex cellular heterogeneity of the kidney and the “insidious” nature of renal fibrosis progression pose unique challenges for identifying appropriate therapeutic targets. Currently, no effective clinical interventions are available for renal fibrosis.

Recently, the ubiquitin–proteasome system has been recognized as a key regulator of kidney homeostasis. In particular, deubiquitinating enzymes (DUBs) serve as “molecular surgeons” in the tubular and interstitial compartments of the kidney, where they remove ubiquitin chains from substrates to protect key regulators from ubiquitin-mediated degradation [3]. Ubiquitin-specific proteases (USPs), such as USP11, USP22, and USP7, which are the largest subfamily of DUBs, perform regulatory roles in various signaling pathways across different types of kidney cells [4–6]. USP7, also known as herpesvirus-associated ubiquitin-specific protease, is a critical member of USPs in eukaryotes. USP7 binds to multiple substrates, protecting them from ubiquitin-mediated degradation, and participates in tumorigenesis by regulating the stability of key oncoproteins and tumor suppressors, including MDM2, p53, N-MYC, PTEN, and DNMT1 [7, 8]. A recent study reported that USP7 promotes G2/M phase arrest in renal tubular epithelial cells by stabilizing checkpoint kinase 1 (CHK1), thereby promoting renal fibrosis [9]. However, to date, no in vivo study has yet confirmed the role of USP7 in renal fibrosis. Moreover, although all renal cell types are implicated in the fibrotic process, the activation of fibroblasts into myofibroblasts is a key contributor [2, 10, 11], and the role of USP7 in renal fibroblast activation remains unclear.

Multiple signaling pathways regulated by USP-mediated ubiquitination, including the TGF-β/Smad, Wnt/β-catenin, and mammalian target of rapamycin (mTOR) pathways, are involved in renal fibroblast activation [12]. The mTOR kinase exists in two distinct protein complexes, namely mTOR complex 1 (mTORC1) and mTOR complex 2 (mTORC2), each with unique structures and functions [13–15]. Numerous studies have demonstrated that the mTORC1 signaling pathway plays a pivotal role in various severe kidney diseases, including fibrosis, by promoting fibroblast activation [16–21]. mTORC1 is negatively regulated by tuberous sclerosis complex 1 (TSC1), and *Tsc1* gene knockout activates mTORC1 signaling, thereby exacerbating renal fibrosis [20]. However, to date, no studies have reported whether USPs directly or indirectly regulate the TSC1/mTORC1 pathway through modification by deubiquitination during fibroblast activation.

Histone modification, an epigenetic modification, that reversibly alters gene expression and cell behavior by modifying histones, serves as a key regulatory mechanism in renal fibrosis [22–25]. Lysine-specific demethylase 5B (KDM5B), a member of the histone lysine demethylase family (KDMs) [26], catalyzes the demethylation of histone H3K4me3 (histone H3 lysine 4 trimethylation) [27–29], thereby suppressing gene expression and regulating various cellular processes, including fibrotic progression [30]. Previous studies have shown that KDM5B reduces histone H3K4me3 modification on the TSC1 promoter, thus inhibiting its transcription and enhancing mTOR signaling activation in postinfarction cardiac repair [31]. Additionally, previous research identified USP7 as a novel deubiquitinating enzyme for KDM5B, which can enhance KDM5B stability [32]. However, the mechanisms underlying the regulation of KDM5B stability during renal fibrosis remain unclear. The interplay between USP-mediated deubiquitination modification and histone methylation in fibroblast activation has also not been explored.

In this study, we investigated the role of USP7 in renal fibrosis, particularly in the activation of renal fibroblasts. We hypothesized that USP7 promotes renal fibrogenesis through the direct or indirect regulation of key downstream pathways through its deubiquitylation activity. Here, we demonstrated the involvement of USP7 in fibroblast activation and renal fibrosis. By combining proteomic sequencing and phosphoproteomic sequencing, we identified the TSC1/mTOR pathway as a key downstream target of USP7, which regulates this pathway mainly by stabilizing KDM5B through deubiquitylation. Our findings reveal a novel epigenetic–post-translational crosstalk mechanism in renal fibrogenesis and implicate USP7 as a potential therapeutic target for preventing renal fibrosis.

## Results

### USP7 is highly upregulated in human CKD biopsy specimens and negatively correlates with estimated glomerular filtration rate

**Fig. 1:**
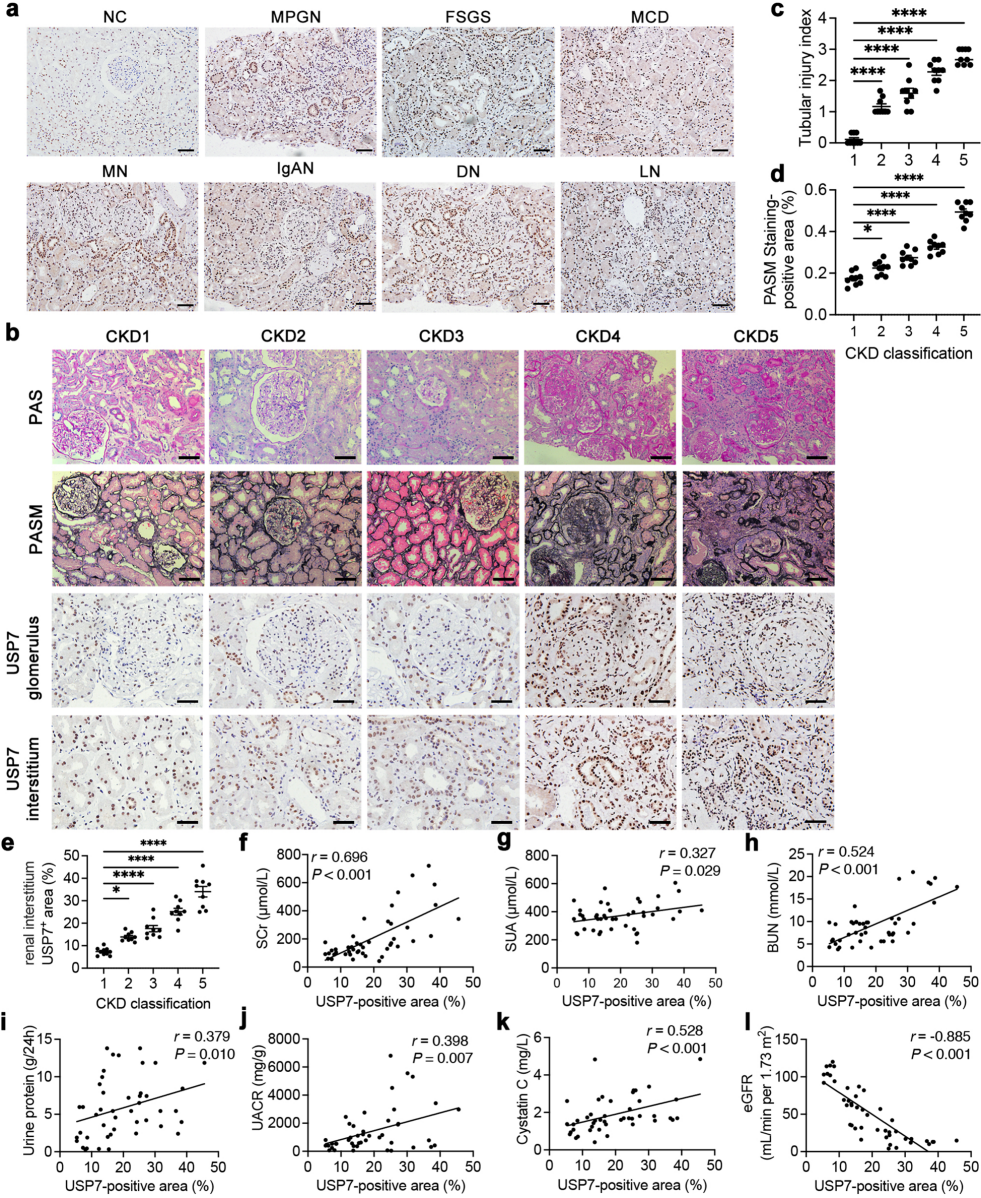
High expression of ubiquitin-specific protease 7 (USP7) in renal biopsy samples from patients with chronic kidney disease (CKD) and its correlation analysis with pathological and clinical parameters. **a** Representative microscopic photographs of immunohistochemical staining for USP7. The samples were from the adjacent normal tissues of renal carcinoma patients (normal control, NC; n = 2 cases), membranoproliferative glomerulonephritis (MPGN; n = 1 case), focal segmental glomerulosclerosis (FSGS; n = 1 case), minimal change nephropathy (MCD; n = 1 case), membranous nephropathy (MN; n = 1 case), IgA nephropathy (IgAN; n = 2 cases), diabetic nephropathy (DN; n = 2 cases), and lupus nephritis (LN; n = 1 case) patients' renal cortical tissues. (Bars = 100 μm). **b** Microscopic photographs of periodic acid-Schiff staining (PAS) (Bars = 100 μm), periodic acid-silver methenamine staining (PASM) (Bars = 100 μm), and immunohistochemical staining for USP7 (Bars = 50 μm) in samples at different CKD stages: CKD stage 1 (n = 9 cases), CKD stage 2 (n = 9 cases), CKD stage 3 (n = 9 cases), CKD stage 4 (n = 9 cases), CKD stage 5 (n = 9 cases). **c** The morphological changes of renal tubular injury were scored according to the PAS staining as described in the Methods section. **d** The positive area of PASM staining in different CKD stages. **e** The positive area of USP7 within the interstitium across different CKD stages. (**f**-**l**) The correlations between the positive area of USP7 and serum creatinine (SCr), serum uric acid (SUA), blood urea nitrogen (BUN), urinary protein, urinary albumin creatinine ratio (UACR), cystatin C, and estimated glomerular filtration rate (eGFR) in all IgA nephropathy patients (n = 45 cases). Data are presented as mean ± standard error. *P < 0.05, **P < 0.01, ***P < 0.001, ****P < 0.0001

To elucidate the role of USP7 in CKD, we conducted immunohistochemical staining to determine USP7 expression in renal biopsy specimens from CKD patients with diverse pathological patterns, including membranoproliferative glomerulonephritis, minimal change nephropathy, membranous nephropathy, IgA nephropathy, focal segmental glomerulosclerosis, diabetic nephropathy, lupus nephritis, and adjacent renal carcinoma tissue (used as the normal control). Notably, USP7 expression was significantly upregulated in these renal biopsy specimens (Fig. 1a). To further clarify the correlation between renal USP7 expression and the severity of renal pathology and fibrosis, we enrolled 45 patients with CKD stages 1–5. Immunohistochemical staining, periodic acid-Schiff (PAS) staining, and periodic acid-silver methenamine (PASM) staining were performed on renal biopsy tissues. The results revealed a significant increase in the positive staining area of USP7 in the tubulointerstitial region and the degree of tubulointerstitial fibrosis with the progression of CKD stage (Fig. 1b-e). Furthermore, USP7 expression exhibited a strong positive correlation with serum creatinine (*r* = 0.696, *P* < 0.001), serum uric acid (*r* = 0.327, *P* = 0.029), serum urea nitrogen (*r* = 0.524, *P* < 0.001), urinary protein (*r* = 0.379, *P* = 0.010), urinary albumin-creatinine ratio (*r* = 0.398, *P* = 0.007), and cystatin C (*r* = 0.528, *P* < 0.001), while demonstrating a negative correlation with estimated glomerular filtration rate (*r* = −0.885, *P* < 0.001) (Fig. 1f-l). These results suggest that renal USP7 expression is significantly upregulated under CKD conditions and is positively correlated with the degree of renal fibrosis.

### USP7 promotes fibrotic progression in NRK-49F cells

**Fig. 2:**
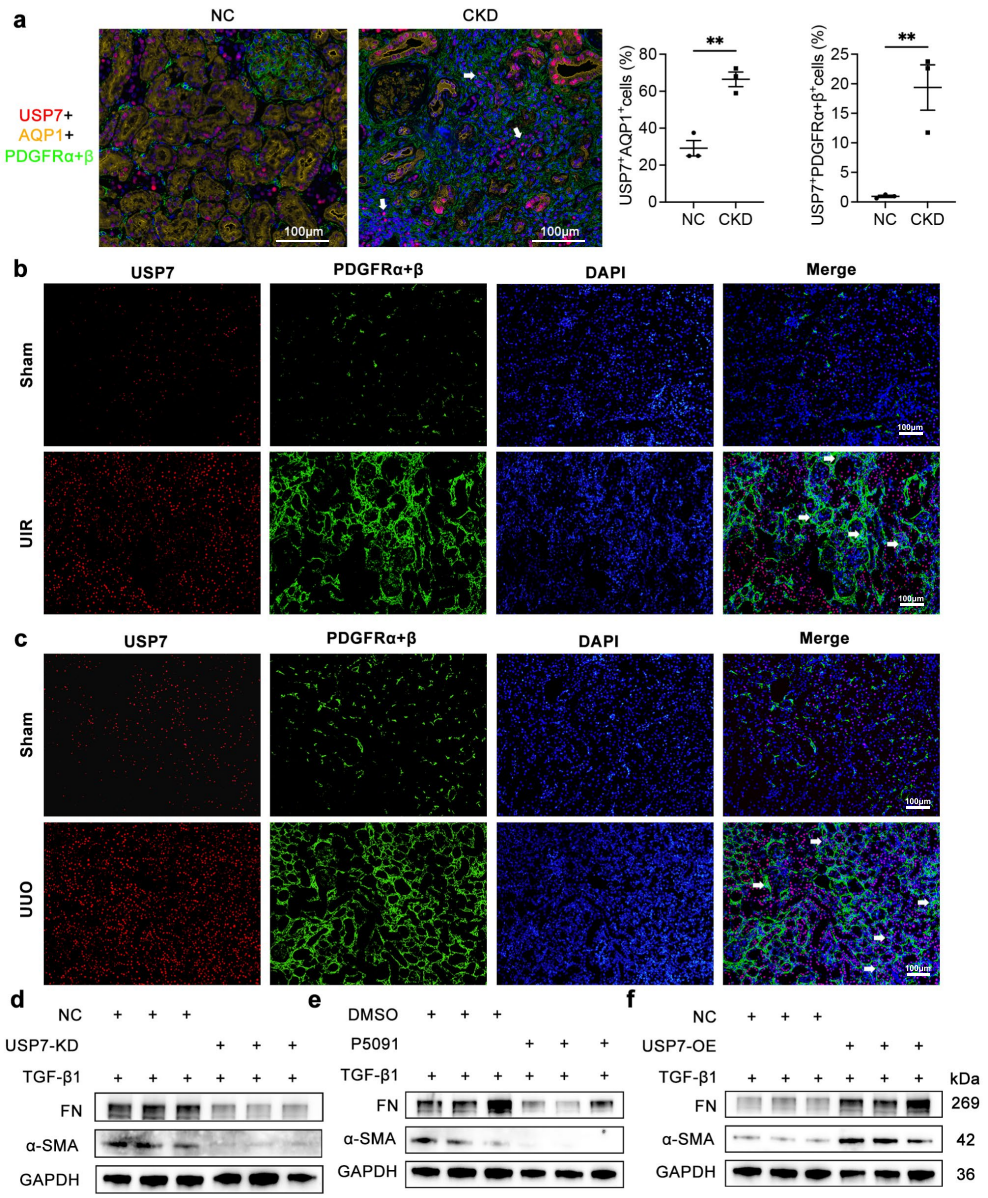
USP7 promotes fibrotic progression in NRK-49F cells. **a** Immunofluorescence staining of renal biopsy tissue from health and CKD patients, labeled by USP7 (red), AQP1 (yellow) and PDFGRα + β (green). The white arrows indicate areas of co-staining of USP7 and PDGFRα + β. n = 3. Bars = 100 μm. **b-c** Microscopic photographs show the co-staining of USP7 and PDGFRα + β in tissue sections of Sham group and UIR group (**b**) or UUO group (**c**). The white arrows indicate areas of co-staining of USP7 and PDGFRα + β. Bars = 100 μm. **d** NC cells and USP7-KD cells were incubated with TGF-β1 (10 ng/ml) for 72 h, and then the cells were collected for immunoblotting analysis. **e** NRK-49F cells were incubated with or without the USP7 inhibitor P5091 (10 μM) under the stimulation of TGF-β1 (10 ng/ml) for 72 h, and then the cells were collected for immunoblotting analysis. **f** NC cells and USP7-OE cells were incubated with TGF-β1 (10 μM) for 72 h, and then the cells were collected for immunoblotting analysis. All data are presented as mean ± SEM. n = 3. **P < 0.01

Previous immunohistochemical analysis results indicated that USP7 upregulation was more pronounced in the interstitium than in tubules. Consequently, immunofluorescence co-staining was performed to examine USP7 expression across different cell types. As shown in Fig. 2a, the numbers of AQP1^+^USP7^+^ cells (USP7^+^ proximal tubular epithelial cells) and PDGFRα + β^+^USP7^+^ cells (USP7^+^ fibroblasts) were significantly increased in renal biopsy tissues from CKD patients, with a more prominent increase observed in PDGFRα + β^+^USP7^+^ cells. Moreover, given that fibroblast activation is a common hallmark of fibrotic diseases, subsequent studies focused on the role of USP7 in fibroblast activation. Next, we examined USP7 expression in renal fibroblasts from two mouse models of kidney fibrosis (unilateral ureteral obstruction [UUO] and unilateral renal ischemia–reperfusion injury [UIR]) and found that USP7 was upregulated in fibroblasts of fibrotic kidneys (Fig. 2b, c). To further investigate the role of USP7 in renal fibroblast activation, an in vitro model was established by stimulating NRK-49F cells with transforming growth factor-β1 (TGF-β1). Immunoblotting analysis demonstrated that TGF-β1 stimulation elevated the levels of fibronectin (FN) and α-smooth muscle actin (α-SMA) in NRK-49F cells, accompanied by increased USP7 expression (Fig. S1a, b). USP7 knockdown (USP7-KD) and USP7 overexpression (USP7-OE) cell models were subsequently established (Fig. S1c, d). Immunoblotting analysis results showed that FN and α-SMA expression was lower in TGF-β1-stimulated USP7-KD cells than in the TGF-β1-stimulated negative control (NC) group (Fig. 2d). Similar results were obtained following treatment with P5091, a small molecular inhibitor of USP7 (Fig. 2e; Fig. S2a, b). Conversely, USP7 overexpression under TGF-β1 stimulation further increased the expression of these fibrotic markers (Fig. 2f). These results collectively suggest that USP7 promotes fibroblast activation.

### USP7 promotes NRK-49F cell fibrotic progression by inhibiting TSC1 expression and activating the mTOR signaling pathway

**Fig. 3:**
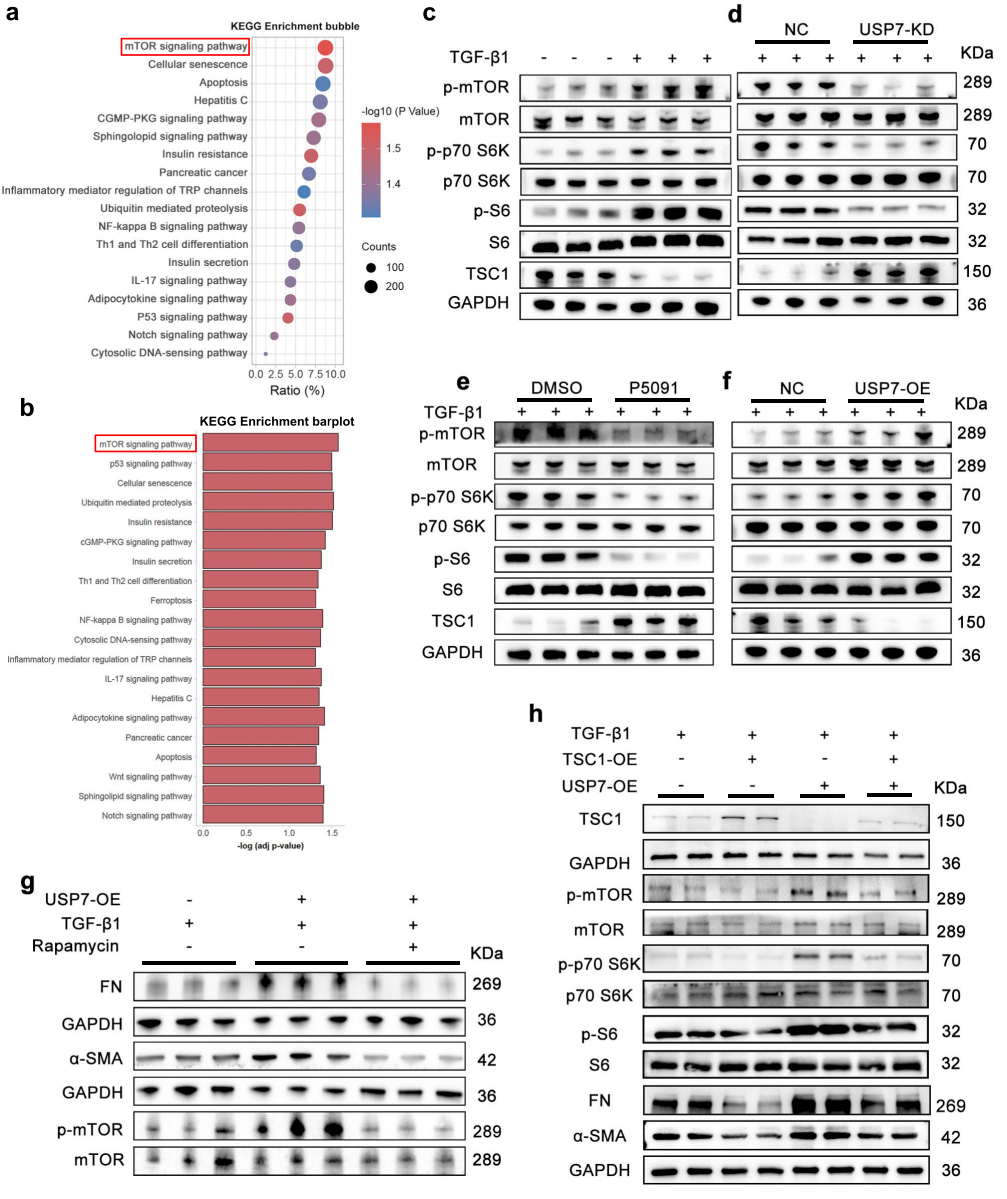
USP7 promotes fibrotic progression in NRK-49F cells by inhibiting TSC1 expression and activating the mTOR signaling pathway. **a** Phosphoproteomic sequencing were performed on USP7-KD and NC stably transfected NRK-49F cells treated with TGF-β1 (10 ng/ml) (with 3 replicates). Kyoto Encyclopedia of Genes and Genomes (KEGG) analysis was performed on all phosphorylated protein sites with altered expression in the form of a bubble chart to clarify the main signaling pathways and other metabolism-related situations in which these phosphorylated proteins are involved. **b** Kyoto Encyclopedia of Genes and Genomes (KEGG) analysis was performed on all phosphorylated protein sites with altered expression in the form of a clustered column chart to clarify the main signaling pathways and other metabolism-related situations in which these phosphorylated proteins are involved. **c** NRK-49F cells were incubated with or without TGF-β1 for 72 h and then subjected to immunoblotting analysis. **d** USP7-KD and NC stably transfected NRK-49F cells were incubated with TGF-β1 and then subjected to immunoblotting analysis. **e** NRK-49 cells were incubated with or without P5091 (10 μM) treatment under the stimulation of TGF-β1 and then subjected to immunoblotting analysis. **f** USP7-OE and NC stably transfected NRK-49F cells were incubated with TGF-β1 and then subjected to immunoblotting analysis. **g** Following a 72-h treatment with rapamycin (100 nM) and TGF-β1, both parental NRK-49F cells and USP7-OE cells were harvested for Western blot analysis. **h** USP7-OE and NC stably transfected NRK-49F cells were transfected with TSC1 overexpression plasmid or scramble plasmid and then treated with TGF-β1 (10 ng/ml) for 72 h. n = 3

To elucidate the mechanism through which USP7 regulates fibrotic progression in NRK-49F cells, USP7-KD cells were subjected to proteomic and phosphoproteomic sequencing (Fig. S3a-f). Phosphoproteomic Kyoto Encyclopedia of Genes and Genomes (KEGG) enrichment analysis suggested that several key cellular processes, including apoptosis, inflammation, and immune cell differentiation, were substantially upregulated in the control group compared to those in the USP7-KD group. Notably, the activation of the mTOR signaling pathway, which is closely associated with renal fibrosis, was markedly enhanced (Fig. 3a, b). However, proteomic analysis revealed no significant changes in the upstream regulators of the mTOR pathway (including TSC1/TSC2, PTEN, FAK, Akt, etc.) following USP7 knockdown (data not shown). Given the inherent variability of proteomic data and the fact that TSC1, a core regulator of the mTOR pathway, is subject to ubiquitination-mediated regulation [33], its protein expression was examined by Western blotting (WB) assay. Unexpectedly, we observed that USP7 knockdown reversed the TGF‑β1‑induced downregulation of TSC1 expression (Fig. 3c, d). Furthermore, USP7 knockdown reduced the phosphorylation levels of mTOR, p70 S6K, and S6, indicating that USP7 knockdown significantly attenuated TGF‑β1‑induced activation of the mTOR pathway (Fig. 3c, d). Consistent results were obtained with the USP7 inhibitor P5091, whereas USP7 overexpression exacerbated TGF‑β1‑induced TSC1 downregulation and mTOR pathway activation (Fig. 3e, f). mTOR Inhibition by rapamycin abrogated the exacerbation of fibrosis induced by USP7 overexpression, suggesting that USP7 mediates fibrogenesis by activating the mTOR signaling pathway (Fig. 3g). Additionally, TSC1 overexpression inhibited USP7 overexpression-induced mTOR pathway activation, and alleviated fibrosis (Fig. 3h). Collectively, these findings suggest that USP7 promotes fibrotic progression in NRK-49F cells by inhibiting TSC1 and thereby activating the mTOR signaling pathway.

### USP7 inhibits TSC1 expression by regulating KDM5B to reduce the histone H3K4me3 modification level at the *Tsc1* gene promoter

**Fig. 4:**
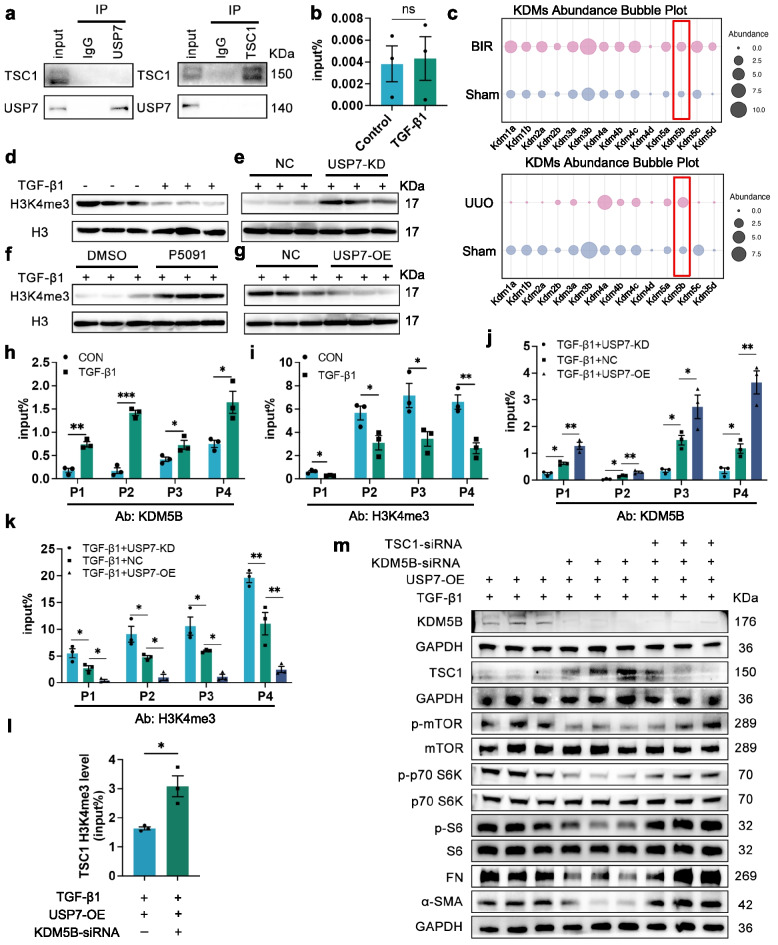
USP7 promotes fibrotic progression in NRK-49F cells by inhibiting TSC1 expression and activating the mTOR signaling pathway. **a** NRK-49F cells were collected and Co-IP experiments were performed using immunoglobulin G (IgG) and antibodies against USP7 or TSC1. **b** The binding of USP7 to the TSC1 promoter region was detected by ChIP-qPCR in NRK-49F cells treated with or without TGF-β1 (10 ng/ml). **c** The expression of all proteins in the histone H3 lysine 4 demethylase family (KDMs) in the GSE254440 database in the BIR, UUO and Sham groups was presented in the form of a bubble chart. **d-g** NRK-49F cells were incubated with or without TGF-β1, and total H3K4me3 levels were assessed by immunoblotting under the following conditions: **d** basal TGF-β1 stimulation; **e** USP7-KD versus negative control (NC) in the presence of TGF-β1; **f** treatment with or without the USP7 inhibitor P5091 (10 μM) in the presence of TGF-β1; **g** USP7-OE versus NC in the presence of TGF-β1. **h** and **i** ChIP-qPCR analysis was performed on the enrichment of KDM5B (**h**) or the modification of histone H3K4me3 (**i**) at the promoter of *Tsc1* in NRK-49F cells with or without TGF-β1 stimulation. **j** and **k** ChIP-qPCR analysis was performed on the enrichment of KDM5B (**j**) or the modification of histone H3K4me3 (**k**) at the *Tsc1* promoter in USP7-KD, USP7-OE, and NC stably transfected NRK-49F cells under the stimulation of TGF-β1. **l** USP7-OE stably transfected NRK-49F cells were transfected with KDM5B siRNA or scramble siRNA in the presence of TGF-β1. The levels of H3K4me3 at *Tsc1* promotor was detected by ChIP-qPCR. **m** KDM5B siRNA and USP7 siRNA was used to transfect USP7-OE stably transfected NRK-49F cells. After treatment with TGF-β1 for 72 h, all cells were collected for protein immunoblotting analysis. All data are presented as mean ± SEM. n = 3. *P < 0.05, **P < 0.01, ***P < 0.001

To determine whether TSC1 undergoes deubiquitylation by USP7, we examined whether USP7 directly interacts with TSC1. However, co-immunoprecipitation (Co-IP) assay revealed no interaction between USP7 and TSC1, and chromatin immunoprecipitation-quantitative polymerase chain reaction (ChIP-qPCR) analysis indicated that USP7 did not directly bind to the *Tsc1* promoter region (Fig. 4a, b). Consequently, we hypothesized that USP7 regulates TSC1 expression through other intermediate factors. Given that TSC1 transcription is regulated by histone methylation modification and that USP7 modulates the ubiquitination of histone demethylases [32], we screened for candidate histone demethylases using published transcriptome sequencing datasets (GSE254440). KDM5B expression was significantly increased in both UUO and bilateral renal ischemia–reperfusion injury (BIR) groups compared to that in the sham-operated group, and KDM5B alone exhibited an elevated expression in both models relative to the sham-operated group (Fig. 4c). Immunoblotting analysis demonstrated that TGF-β1 stimulation reduced total histone H3K4me3 levels. Notably, both genetic knockdown of USP7 and its inhibition by P5091 reversed this reduction, whereas USP7 overexpression potentiated the decrease. (Fig. 4d-g). ChIP-qPCR analysis showed that TGF-β1 stimulation increased the recruitment of KDM5B to the *Tsc1* gene promoter (Fig. 4h) and decreased histone H3K4me3 modification level at this locus (Fig. 4i). In USP7-KD and USP7-OE cells subjected to TGF-β1 stimulation, USP7 knockdown significantly reduced the recruitment of KDM5B to the *Tsc1* gene promoter and substantially increased the histone H3K4me3 modification level at the promoter, whereas USP7 overexpression yielded opposite effects (Fig. 4j, k). These results suggest that USP7 modulates the recruitment level of KDM5B at the *Tsc1* gene promoter, thereby reducing the histone H3K4me3 modification level at the promoter.

To further confirm the role of KDM5B in these effects, we knocked down KDM5B in USP7-KD cells. This intervention significantly increased H3K4me3 levels at the *Tsc1* gene promoter and markedly elevated TSC1 protein expression, accompanied by mTOR pathway inhibition and fibrosis alleviation (Fig. 4l, m). Conversely, additional knockdown of TSC1 reversed both mTOR inhibition and fibrosis alleviation (Fig. 4m).

In summary, these results demonstrate that USP7 inhibits TSC1 and activates the mTOR pathway through KDM5B-mediated H3K4me3 modification at the *Tsc1* gene promoter.

### USP7 promotes the stability of the KDM5B protein in NRK-49F cells by deubiquitinating KDM5B

**Fig. 5:**
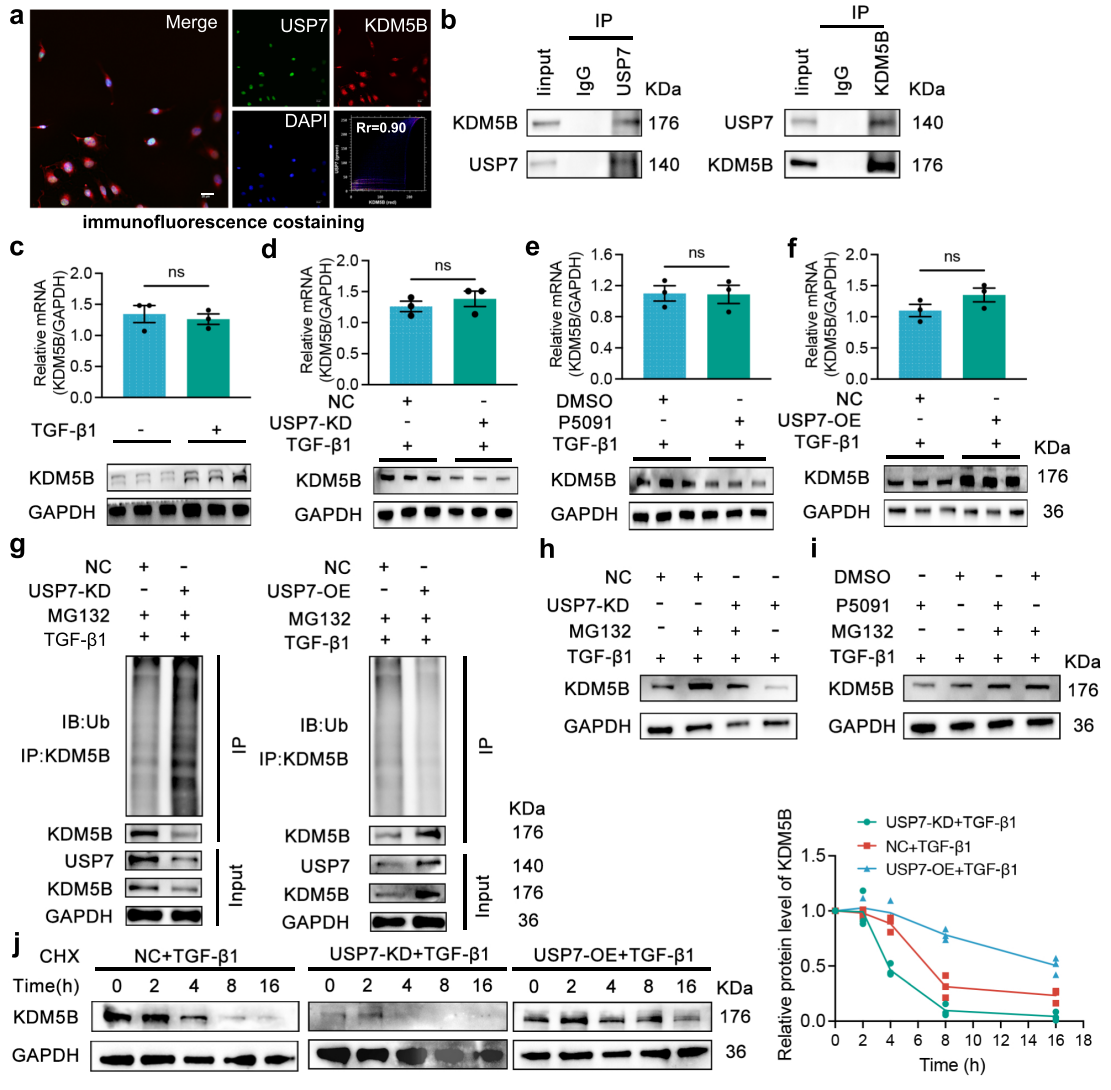
USP7 promotes the stability of KDM5B protein in NRK-49F cells by deubiquitinating KDM5B. **a** Microscopic photographs show the immunofluorescence co-staining of USP7 and KDM5B in NRK-49F cells. Bars = 20 μm. Pearson correlation analysis was used to determine statistical significance, with *Rr* = 0.90. **b** Interaction between USP7 and KDM5B detected by CO-IP. **c**-**f** mRNA and protein expression of KDM5B in NRK-49F cells under the following conditions: **c** basal TGF-β1 stimulation; **d** USP7-KD versus NC in the presence of TGF-β1; **e** treatment with or without the USP7 inhibitor P5091 (10 μM) in the presence of TGF-β1; **f** USP7-OE versus NC in the presence of TGF-β1. **g** USP7-KD, USP7-OE, and NC cells were incubated with TGF-β1, and MG132 was added 4 h before collection. Then, cell lysates were collected, and immunoprecipitation was performed using KDM5B antibody, followed by ubiquitin immunoblotting (IB) analysis. All input lysates for immunoprecipitation were analyzed by immunoblotting against USP7, KDM5B, and GAPDH. **h** USP7-KD and NC stably transfected NRK-49F cells were treated with TGF-β1, and then treated with MG132 for 4 h. **i** Under the stimulation of TGF-β1, NRK-49F cells were treated with or without the USP7 inhibitor P5091, and then treated with MG132 for 4 h. **j** USP7-KD, USP7-OE, and NC stably transfected NRK-49F cells treated with TGF-β1, and then treated with cycloheximide (CHX). All cells were collected at different time points for protein immunoblotting analysis. A statistical line chart of the half-life of the KDM5B protein was drawn. All data are presented as mean ± SEM. n = 3. “ns” indicates no significant difference

Given that KDM5B is deubiquitinated and stabilized by USP7 during nasopharyngeal carcinoma progression [32], we investigated the regulatory role of USP7 on KDM5B in the pathogenesis of fibrosis. The Ubiquitin Browser indicated a close association between USP7 and KDM5B (Fig. S4a). Moreover, analysis of the amino acid sequence of KDM5B revealed an overlap between the USP7-binding motif and the conserved region of KDM5B (Fig. S4b). Subsequently, the HDOCK program predicted an interaction between USP7 and KDM5B proteins, with a protein–protein docking score of 256.26. This interaction involved multiple amino acids, with the main interaction types being hydrogen bonds and salt bridge (Table S2, Fig. S4c, d). Immunofluorescence staining of NRK-49F cells also demonstrated co-localozation of USP7 and KDM5B, with a correlation coefficient *Rr* = 0.90 (Fig. 5a). Co-IP experiments confirmed that endogenously expressed KDM5B interacted with USP7 in NRK-49F cells (Fig. 5b). Following TGF-β1 stimulation, the KDM5B protein expression level increased, while its mRNA level remained unchanged (Fig. 5c). Moreover, KDM5B protein levels were significantly decreased following genetic knockdown or pharmacological inhibition of USP7, but markedly increased after USP7 overexpression. In contrast, KDM5B mRNA levels remained unchanged under all these conditions (Fig. 5d-f). To elucidate the mechanism through which USP7 regulates the KDM5B protein, we assessed the endogenous ubiquitination levels of KDM5B protein under USP7 knockdown or overexpression condition. USP7 knockdown increased the polyubiquitination of KDM5B, while USP7 overexpression decreased its polyubiquitination (Fig. 5g). Additionally, USP7 knockdown or P5091 treatment reduced KDM5B protein expression, and this reduction could be prevented by the proteasome inhibitor MG132 (Fig. 5h, i). Moreover, following USP7 knockdown or inhibition, the protein half-life of KDM5B was significantly shortened, whereas USP7 overexpression yielded the opposite effect (Fig. 5j). Collectively, these results indicate that USP7 stabilizes KDM5B through its deubiquitylation.

### Fibroblast-specific depletion or pharmacological inhibition of USP7 alleviates UIR- and UUO- induced renal fibrosis

**Fig. 6:**
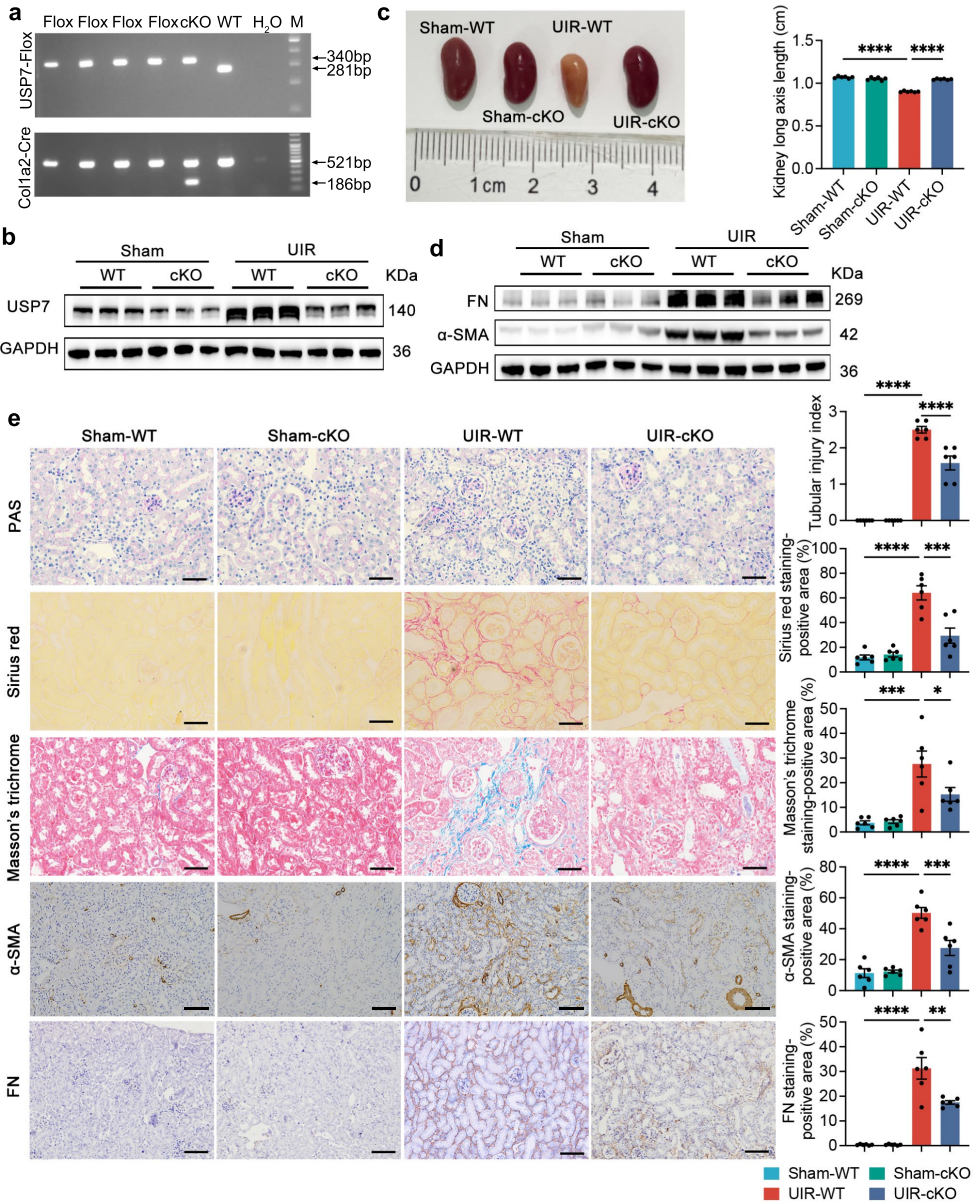
Fibroblast-specific depletion of USP7 alleviates renal fibrosis induced by unilateral renal ischemia–reperfusion injury (UIR). **a** A fibroblast-specific depletion model of ubiquitin-specific protease 7 (USP7) mice (*Col1a2*-Cre^+^*Usp7*.^flox/flox^ mice) was constructed. Gene typing was confirmed by tail sample preparation and PCR when the mice were 2 weeks old. wild-type (WT) mice and conditional knockout (cKO) of USP7 mice were used to induce renal fibrosis by UIR. **b** Renal protein level of USP7 in each group. **c** Gross images of mouse kidneys. **d** Renal protein levels of α-SMA and Fibronectin (FN) in each group. **e** Microscopic photographs show the PAS staining (Bars = 50 μm), Masson’s trichrome staining (Bars = 50 μm), Sirius red staining (Bars = 50 μm), and immunohistochemical staining (Bars = 100 μm) results of α-SMA and FN of the kidneys. The morphological changes of renal tubular injury were scored according to the PAS staining as described in the Methods section. The other four pictures show the positive areas of Masson’s trichrome (blue), Sirius red (red), and immunohistochemical staining of α-SMA and FN. All data are presented as mean ± SEM. n = 6. *P < 0.05, **P < 0.01, ***P < 0.001, ****P < 0.0001

To elucidate the role of USP7 in renal fibrosis progression, we established a fibroblast-specific USP7 depletion model using *Col1a2*-Cre^+^*Usp7*^flox/flox^ mice (Fig. 6a, b). These mice were subsequently subjected to UUO or UIR surgery to induce renal fibrosis. At 14 days post-UIR induction, the kidneys of USP7- wild-type (WT) mice exhibited a loss of luster, showed signs of shrinkage, and became markedly rigid (Fig. 6c). Immunoblotting analysis and immunohistochemical staining confirmed a marked increase in FN and α-SMA expression in the kidneys of USP7-WT mice (Fig. 6d, e). PAS staining, Masson’s trichrome staining, and Sirius red staining further revealed prominent glomerulosclerosis and tubulointerstitial fibrosis in the kidneys of UIR-WT mice, accompanied by tubular dilation, epithelial atrophy, and loss of the brush border. However, *Usp7* gene deletion significantly mitigated these pathological alterations (Fig. 6e). Similar findings were observed in the UUO model (Fig. S5). Subsequently, we further validated the role of USP7 in renal fibrosis using the specific inhibitor P5091. The results showed that P5091 significantly attenuated renal fibrosis in UUO and UIR mice (Fig. S6). Given that high USP7 expression correlated with renal dysfunction in CKD patients (Fig. 1), we further validated these findings using the BIR model. Fibroblast-specific ablation of USP7 markedly ameliorated renal injury, as evidenced by reduced serum creatinine (SCr), diminished renal expression of kidney injury molecule 1 (KIM‑1) and neutrophil gelatinase-associated lipocalin (NGAL), and alleviated fibrosis in BIR mice (Fig. S7). Taken together, these results underscore the critical role of USP7 in renal interstitial fibrosis and kidney dysfunction.

### USP7 conditional knockout and P5091 treatment inhibit KDM5B expression, restore TSC1 protein level, and suppress mTOR signaling pathway activation in UIR and UUO mouse models

**Fig. 7:**
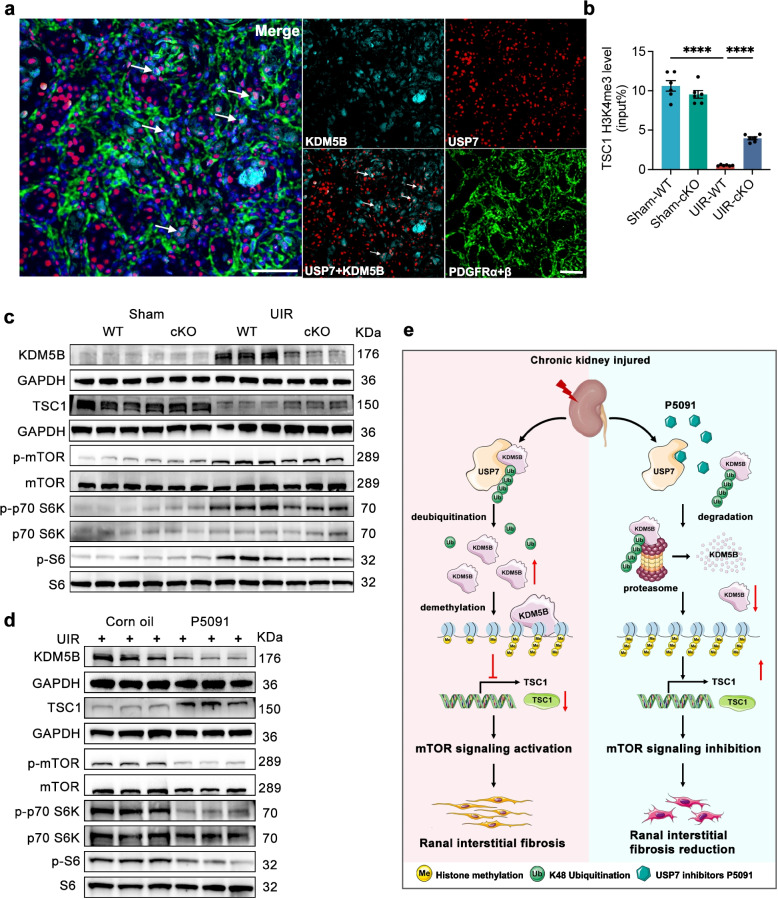
USP7 conditional knockout and P5091 intervention inhibit KDM5B expression, restore TSC1 protein level, and inhibit mTOR signaling pathway activation in UIR mouse models. **a** Microscopic photographs display the immunofluorescence co-staining of USP7, KDM5B, and platelet-derived growth factor receptor α and β (PDFGRα + β) in damaged kidneys. The white arrows indicate the co-stained cells. Bars = 50 μm. **b** H3K4me3 levels at *Tsc1* promotor in each group. **c** Renal protein expression in cKO mice and WT mice receiving sham or UIR operation. **d** Renal protein expression in WT mice receiving P5091 administration after UIR operation. **e** The regulatory mechanism of USP7 in renal interstitial fibrosis. USP7 enhances the stability of KDM5B in renal fibroblasts through deubiquitylation, removes H3K4me3 mark of the *Tsc1* promoter through KDM5B, potentiates the activation of mTOR signaling pathway transduction, and promotes the progression of renal fibrosis. Treatment with the inhibitor P5091 of USP7 can alleviate this process. All data are presented as mean ± standard deviation. n = 6. ****P < 0.0001

To further validate the previously identified mechanism by which USP7 alleviates fibrosis in vivo, we examined the KDM5B/TSC1/mTOR pathway in conditional knockout (cKO) and P5091-treated mice subjected to UIR and UUO modeling procedures. First, immunofluorescence staining revealed co-localization of USP7 and KDM5B in fibroblasts of the injured kidneys (Fig. 7a). Second, ChIP-qPCR analysis indicated that fibroblast-specific depletion of USP7 reversed the decrease in histone H3K4me3 modification level at the *Tsc1* promotor in UIR and UUO mice (Fig. 7b, Fig. S8a). Third, immunoblotting analysis demonstrated that, compared to WT mice, USP7-cKO mice exhibited significantly reduced KDM5B expression, restored TSC1 expression, and decreased phosphorylation levels of mTOR, p70S6K, and S6, indicating inhibition of the mTOR signaling pathway (Fig. 7c, Fig. S8b). Given the identical inhibitory effect of P5091 on the KDM5B/TSC1/mTOR pathway (Fig. 7d, Fig. S8c), the findings suggest that USP7 promotes renal fibrosis progression by modulating the KDM5B/TSC1/mTOR axis. Collectively, we demonstrate that USP7 enhances the stability of KDM5B in renal fibroblasts by deubiquitination, eliminates histone H3K4me3 modification at the *Tsc1* gene promoter through KDM5B, potentiates mTOR pathway signal transduction activation, and promotes renal fibrosis progression. Consequently, inhibiting USP7 function represents a potential therapeutic approach for ameliorating renal fibrosis (Fig. 7e).

## Discussion

Emerging evidence indicates that USPs play a significant role in renal fibrogenesis by deubiquitinating key factors involved in pathways such as profibrotic signaling, inflammatory responses, and oxidative stress [34–36]. USP7, a member of the USP family, is known for its functions in cell growth [37], proliferation [38], differentiation [39], and epigenetic modification [40]. A recent study reported that USP7 activates the TGF-β1 signaling pathway by stabilizing Smad2/3, thereby contributing to pulmonary fibrosis [41]. However, its pathological function in renal fibrosis remains poorly understood. In this study, we identified USP7 as a crucial regulator of renal fibrosis during chronic kidney injury progression. Consistent with Shao’s findings that USP7 expression is positively associated with TGF-β levels in patients with calculi related CKD and promotes renal fibrosis [9], our study also demonstrated a significant increase in renal USP7 expression in CKD patients, with a positive correlation between USP7 expression and the fibrotic area. It is undeniable that different CKD etiologies may influence USP7 expression and fibrosis. Various metabolic and immune disorders may elevate USP7 expression, and USP7 subsequently participates in regulating these disorders, such as lupus nephritis and diabetes [42, 43]. Furthermore, oxidative stress and inflammation promote USP7 expression [44, 45], and the levels of these factors vary among different CKD populations. To minimize the influence of metabolic and autoimmune factors on USP7, we used UUO and UIR models and demonstrated upregulated expression of USP7 in fibrotic kidneys, which promoted fibrosis progression.

We observed that USP7 expression was upregulated in both tubular epithelial cells and fibroblasts in fibrotic kidneys, with a more prominent increase in fibroblasts. Therefore, we generated fibroblast-specific USP7 knockout mice and confirmed that USP7 promotes fibroblast activation. Previous studies have revealed that USP7 upregulation in renal tubular epithelial cells after kidney injury influences the cell cycle, thereby promoting epithelial–mesenchymal transition (EMT) and renal fibrosis. However, we also observed relatively high basal expression level of USP7 in normal kidneys. Given that the deubiquitinating activity of most USPs depends on “activation on demand” (including binding to specific co-factors or post-translational modifications) [46–48], and that USP7 is activated by phosphorylation [49], further investigations are required to confirm whether the highly expressed USP7 in uninjured tubular epithelial cells is functionally active and elucidate the mechanisms through which it becomes activated after injury.

To investigate whether USP7 modulates fibroblast activation through post‑transcriptional regulation of key factors involved in fibrosis‑related pathways, we performed proteomic and phosphoproteomic analyses to screen for downstream targets of USP7. We identified the TSC1/mTOR pathway as an essential downstream target of USP7. As a key component of the mTOR complex, mTORC1 is involved in various kidney diseases. In fibrotic kidneys, the mTORC1 signaling pathway is activated and promotes fibroblast activation. TSC1 is a key negative regulator of mTORC1 signaling. Specific knockout of TSC1 in fibroblasts induces renal interstitial fibrosis [20]. Another study demonstrated that TSC1-mediated mTORC1 activation promotes glycolysis in renal tubular epithelial cells, thus facilitating the progression of interstitial fibrosis in renal fibrosis [50]. These findings were further verified in both in vivo and in vitro experiments. Our results indicate that USP7 promotes fibrotic progression in part by inhibiting TSC1 expression and activating the mTOR pathway. While TSC1 is a major downstream effector mediating this pathway, we acknowledge that other USP7-regulated signaling routes may also contribute to fibrogenesis. Nevertheless, targeting USP7 or the TSC1-mTOR axis remains a promising therapeutic strategy for CKD.

Althrough direct deubiquitination of TSC1 by USP7 was not observed in our study, previous studies have established a link between epigenetic modification and the mTORC1 signaling pathway [51]. USP7 can also coordinate histone methylation by stabilizing histone methylases and demethylases [52]. Hence, we investigated whether USP7 regulates TSC1 expression through histone methylation. From previously reported transcriptome sequencing data, we identified the histone demethylase KDM5B, which was highly expressed in the fibrotic kidneys of UUO and BIR models. KDM5B, a member of the JMJD subfamily, transcriptionally represses gene expression by reducing H3K4me3 levels at gene promoters. It has been reported to reduce histone H3K4me3 levels at the TSC1 promoter in cardiac macrophages, thereby inhibiting reparative transformation through the mTOR pathway [31]. Consistently, our results suggest that under fibrotic conditions, KDM5B bound to the *Tsc1* promoter and reduced its histone H3K4me3 levels, thereby downregulating TSC1 and activating mTOR. We also confirmed that USP7 modulated KDM5B binding to the *Tsc1* promoter, which correspondingly influenced histone H3K4me3 levels at this promoter, thus influencing TSC1 transcription and fibrotic progression. Additionally, we found that USP7 directly interacted with KDM5B, and stabilized it through deubiquitinase activity, similar to a recent study which showed that USP7 stabilized KDM5B and promoted tumor progression and cisplatin resistance in nasopharyngeal carcinoma [32]. These findings suggest that KDM5B is an important intermediate mediator of the TSC1/mTOR pathway regulated by USP7, revealing a new epigenetic regulatory pathway of renal fibrosis under pathological stress conditions. However, it should be acknowledged that KDM5B, a histone H3K4 demethylase with broad genomic functions, may regulate additional transcriptional targets beyond TSC1 in renal fibrosis. Notably, KDM5B promotes cardiac fibrosis by repressing activating transcription factor 3 (ATF3) and facilitates pulmonary fibrosis by suppressing PDGFRα/β expression [30, 53]. Nevertheless, the mechanistic role of KDM5B in kidney fibrosis remains unexplored. Future approaches such as ChIP-seq or transcriptomics are warranted to identify other KDM5B targets and elucidate the epigenetic network driving fibroblast activation.

While these findings are encouraging, several limitations of this study must be acknowledged. First, given that fibroblast activation represents a central event in fibrosis, our study focused solely on the role of USP7 in renal fibroblast activation. Although a recent study has implicated USP7 in the EMT of renal tubular epithelial cells, further research is required to reveal and validate its function and mechanisms in these cells. Second, although both in vitro and in vivo data demonstrated that the KDM5B/TSC1/mTOR axis mediated USP7‑promoted renal fibrosis, this pathway is not exclusive. Further studies are required to determine whether USP7 promotes renal fibrosis by deubiquitinating other epigenetic regulators or regulating other pro‑fibrotic signaling pathways. Third, although in vivo evidence indicates that P5091, an inhibitor of USP7, attenuates renal fibrosis by inhibiting the KDM5B/TSC1/mTOR axis, its side effects, including off-target effects or regulation of other proteins or pathways, along with the lack of an effective therapeutic dose, pose significant obstacles to its clinical application.

In summary, using gene knockout or pharmacological inhibition methods, we have for the first time demonstrated that USP7 is upregulated in activated renal fibroblasts and it may act as a key epigenetic regulator during renal fibrosis progression. Mechanistically, USP7 mediates the reduction of histone H3K4me3 modification at the *Tsc1* promotor by stabilizing KDM5B, leading to the activation of the mTOR signaling pathway and fibroblast activation. Overall, our study provides evidence to support USP7 as a promising therapeutic target for treating renal fibrosis.

## Materials and methods

More comprehensive details of all experimental methods are provided in Supplementary Methods.

### Human renal biopsy samples

Human renal biopsy samples were obtained from patients admitted to the Department of Nephrology, Zhongshan Hospital, Fudan University, between June 2021 and December 2022 during routine clinical diagnostic procedures (Table S1). Control samples were obtained from healthy renal tissues of individuals who underwent nephrectomy for tumors and had no history of diabetes or CKD. Further details regarding human renal biopsy samples are provided in Supplementary Methods.

### Animals and treatment

Male C57BL/6 mice, aged 6 to 8 weeks, were subjected to UUO or UIR surgery to induce renal fibrosis, as described previously [54]. The damaged kidneys were harvested 1 week after UUO surgery and 2 weeks after UIR surgery. P5091 (25 mg/kg) was intraperitoneally administered daily, starting from the day after modeling until kidneys harvest. Detailed information regarding animals and treatment protocols is provided in Supplementary Methods.

### Construction of fibroblast-specific ubiquitin-like PHD and USP7 conditional knockout mice

*Usp7*^flox/flox^ mice and *Col1a2*-Cre/ERT2 transgenic mice were purchased from the Shanghai Model Organisms Center. To generate an inducible fibroblast-specific USP7 conditional knockout mouse model, *Usp7*^flox/flox^ mice were crossed with *Col1a2*-Cre/ERT2 transgenic mice. The Cre-ERT system was activated by oral gavage of tamoxifen (100 mg/kg; Sigma, 10,540–29-1, USA) for 5 consecutive days, starting 1 week prior to surgery in homozygous male mice (*Col1a2*-Cre⁺ *Usp7*^flox/flox^). Following UUO or UIR surgery, tamoxifen was continuously administered daily until the day before sacrifice.

### Proteomics

100 μg of protein samples was collected and digested overnight at 37 °C with trypsin at a 1:50 mass ratio (enzyme: protein). Peptide were quantified using the Thermo Fisher Scientific peptide quantification kit (catalog number 23275), followed by phosphorylation enrichment. Peptide separation was performed using a VanquishNeo (Thermo) chromatography system, and the samples separated by nano-high-performance liquid chromatography were analyzed by mass spectrometry using an Orbitrap Astral mass spectrometer (Thermo, USA). The DIA raw data were imported into the Spectronaut™ 18 software system for database search and analysis. The t.test function in the R language was used to calculate the P value of significance of differences between the groups and the fold change (FC). Proteins with a significant P value of < 0.05 and a fold change greater than 1.2 times were considered differentially expressed proteins.

### Phosphoproteomics

LC–MS/MS analysis was performed using an Orbitrap Astral mass spectrometer (Thermo, USA) coupled with a VanquishNeo liquid system (Shanghai Majorbio Bio- Pharmaceutical Technology Co., Ltd.). The DIA raw data were imported into the Spectronaut™ software system for database search and analysis. A new variable termed protein phosphorylation state value (ΔPvalue) was defined to measure the differential changes of proteins based on the upregulation and downregulation of the expression levels of phosphorylated peptides in the protein and the number of phosphorylated peptides. The ΔPvalue was calculated by summing the log2FC values of all the differential phosphorylated peptides corresponding to the same protein. A ΔPvalue of > 1 indicated upregulation of phosphorylated peptides in that protein. The Kyoto Encyclopedia of Genes and Genomes (KEGG) pathway database (http://www.genome.jp/kegg//) was used to analyze the metabolic pathways involved in the differential proteins.

### Protein–protein interaction prediction

To predict the interaction between USP7 and KDM5B, we employed the HDOCK server. HDOCK uses a hybrid docking strategy combining template-based modeling and ab initio free docking, enabling robust and accurate prediction of protein–protein complexes. USP7 and KDM5B structures were modeled using AlphaFold3. Docking was performed with default parameters, generating 100 initial poses. The model with the lowest docking energy was selected as the most likely binding conformation. Visualization and structural analysis of the docked complex were performed using PyMOL (v.3.0.3).

### Quantification and statistical analysis

All data are expressed as mean ± standard error of the mean (SEM). Student's t-test was used for comparisons between two groups, and one-way analysis of variance (ANOVA), followed by Tukey’s post-hoc test, was applied for comparison involving three or more groups. All statistical analyses were performed using GraphPad Prism v.9.1.1. All tests were two-tailed, and a *P*-value of < 0.05 was considered statistically significant.

## Supplementary Information

Supplementary Material 1.

## Data Availability

The raw proteomics and phosphoproteomics data generated in this study have been deposited into the ProteomeXchange Consortium via the PRIDE partner repository with the dataset identifier PXD059829. The RNA-seq datasets for UUO mouse kidneys are available in the Gene Expression Omnibus under accession code GSE254440 (https://github.com/CostaLab/scopen). Data for Figure S4a were generated using the Ubiquitin Browser (http://ubibrowser.bio-it.cn/), and data for Figure S4c, d were obtained from the HDOCK web server (http://hdock.phys.hust.edu.cn/).
