# Supplementary material for: Deubiquitinase USP7 stabilizes the histone demethylase KDM5B and promotes the progression of renal fibrosis through the TSC1/mTOR axis
Source: Mol Biomed. 2026 Aug 5;7:126. doi: 10.1186/s43556-026-00531-3 (PMC13442801; doi:10.1186/s43556-026-00531-3)
Supplement: Supplementary file 1 [file 43556_2026_531_MOESM1_ESM.pdf]

Deubiquitinase USP7 stabilizes the histone demethylase KDM5B and promotes the progression of renal fibrosis through the TSC1/mTOR axis

Shiqi Lv[1 2 3 4†], Ziyan Shen[1 2 3 4†], Yulu Gu[5†], Yulin Wang[1 2 3 4], Jiayi Wang[1 2 3 4], Han Zhang[1 2 3 4], Hong Liu[1 2 3 4], Jing Chen [1 2 3 4], Cheng Zhu[1 2 3 4], Xinhui Huang [1 2 3 4], Tongqiang Liu[5*], Xiaoqiang Ding[1 2 3 4*], Xiaoyan Zhang[1 2 3 4*]

1 Department of Nephrology, Zhongshan Hospital, Fudan University, Shanghai, 200032, China.

2 Shanghai Medical Center of Kidney Disease, Shanghai, 200032, China.

3 Shanghai Key Laboratory of Kidney and Blood Purification, Shanghai, 200032, China.

4 Shanghai Institute of Kidney and Dialysis, Shanghai, 200032, China.

5 Department of Nephrology, The Second People's Hospital of Changzhou, the Third Affiliated Hospital of Nanjing Medical University, Jiangsu, 213001, China.

# Contributed equally.

Competing Interests: None declared.

Corresponding author:

*Xiaoyan Zhang, Kidney and Dialysis Institute of Shanghai, No. 136 Medical College Road, Shanghai 200032, China. E-mail: zhang.xiaoyan@zs-hospital.sh.cn; Tel. / fax: +86-21-64038472

*Xiaoqiang Ding, Department of Nephrology, Zhongshan Hospital, Fudan

University, No. 180 Fenglin Road, Shanghai 200032, China. E-mail: ding.xiaoqiang@zs-hospital.sh.cn; Tel. / fax: +86-21-64038472
*Tongqiang Liu, Department of Nephrology, The Second People's Hospital of Changzhou, the Third Affiliated Hospital of Nanjing Medical University, Jiangsu, 213001, China. E-mail: liuyf1106@126.com; Tel. / fax: 0519—81099988

Supplementary Materials and Methods

Human renal biopsy samples

Renal biopsies were performed as part of routine clinical diagnostic investigations and collected as outlined in Supplementary Table S1. The present study enrolled 45 CKD patients aged between 18 and 70 years who had received renal biopsy at the Department of Nephrology, Zhongshan Hospital, Fudan University, between June 2021 and December 2022. Patients with malignant tumors, organ transplantation, chronic infectious diseases, or prior receipt of dialysis, corticosteroids, or immunosuppressive agents were excluded. Control samples were sourced from the healthy kidney poles of individuals undergoing tumor nephrectomies who had no diabetes or CKD. Relevant images of PAS and PASM staining across CKD stages 1 to 5 were retrieved from the pathology reporting system of Zhongshan Hospital.

Laboratory examination data were obtained from the hospital's case management system.

Animals and treatment

Six to 8-week-old wild-type male C57/BL6 mice (Shanghai Jihui Laboratory Animal Breeding Co., Ltd., China) were housed in the Experimental Animal Center of Fenglin Campus, Fudan University, under a 12-hour light-dark cycle with free access to food and water. All animals were acclimatzed to this environment for 7 days before the experiment. After acclimation, mice were randomly assigned to groups based on cage position, and underwent UUO or UIR surgery to induce renal fibrosis. For UUO surgery, the right ureter was ligated with 4-0 silk thread. For UIR induction, the right renal pedicle was clamped with a microaneurysm clip for 35 minutes while maintaining body temperature at 38°C. In the sham-operated group, the right kidney alone was exposed without further treatment. To investigate the effect of the USP7 inhibitor P5091 on renal fibrosis, mice in both fibrosis model groups received daily intraperitoneal injections of P5091 (S7132, Selleck, Houston, USA) dissolved in corn oil, starting from the day of modeling until the day before kidney collection. Based on concentration gradient results, the final dose was selected as 25 mg/kg (Supplementary Figure S6a). The control group was injected with an equal volume of corn oil. Kidney samples were collected 1 week

after UUO and 2 weeks after UIR for protein analysis and histological examination. For the BIR model, bilateral renal pedicles were clamped for 28 minutes with body temperature maintained at 38°C. Serum samples were collected 14 days after surgery to measure serum creatinine (SCr). This study focused exclusively on male mice; therefore, the applicability of these findings to female mice remains unclear. Future experiments will verify the role of the studied mechanisms in female mice.

Renal pathological staining

Mouse kidneys were fixed in 4% paraformaldehyde, embedded in paraffin, and cut into 4-μm-thick sections for performing PAS staining, Sirius red staining, and Masson's trichrome staining. The kidney sections were dewaxed and subjected to antigen retrieval in heated sodium citrate buffer. Subsequently, the sections were incubated with 3% hydrogen peroxide and 5% bovine serum albumin (BSA). The primary antibodies used were anti-α-SMA (1:1000, A2547, Sigma, USA) and anti-fibronectin (1:1000, ab2413, Abcam, USA). Incubation with secondary antibodies and diaminobenzidine staining were performed using the GTVision III Detection System kit (GK500705, GeneTech, Shanghai, China). Images were captured using an optical microscope. Ten random fields (magnification ×200) were selected from each sample and analyzed using ImageJ software.

Pathological evaluation

To evaluate the degree of renal tubular injury, morphological injuries (epithelial necrosis, intraluminal necrotic debris, and renal tubular dilation) in 10 fields of three sections from each kidney were quantified and scored according to the following grades: no injury = 0 points; injury degree < 30% = 1 point; injury degree between 30–60% = 2 points; injury degree > 60% = 3 points.

Immunohistochemical and immunofluorescence analyses

Mouse kidneys were fixed in 4% paraformaldehyde, embedded in paraffin, and cut into 4-μm-thick sections. After dewaxing and antigen retrieval, the kidney sections were blocked with 3% hydrogen peroxide and 3% BSA for 1 hour to inhibit endogenous peroxidase activity. The primary antibodies used were anti-USP7 (1:200, 4833, Cell Signaling Technology, USA), anti- KDM5B (1:200, MA5-51950, ThermoFisher, USA), and anti- PDGFRα+β (1:200, ab32570, Abcam, USA). Cell nuclei were stained with DAPI. Images were captured using an Olympus FV3000 confocal microscope.

Cell culture and treatment

Rat normal renal fibroblasts (NRK-49F cells, ATCC, USA) were cultured in Dulbecco's Modified Eagle Medium (DMEM, 11965092, Gibco, USA) supplemented with 10% fetal bovine serum (FBS, A5670701, Gibco, USA) and

1% penicillin-streptomycin. Before stimulation, NRK-49F cells were starved overnight in DMEM containing 0% FBS. Based on time and concentration gradient results, TGF-β1 (7754-BH, R&D, USA) was used at a concentration of 10 ng/mL for 72 hours (Supplementary Figure S1a). Consistent with the findings reported in the literature, P5091 was administered at a concentration of 10 μM for 72 hours[1]. Prior to collecting ubiquitinated proteins, MG132 (HY-13259, MCE, USA) was used at a concentration of 5 μM for 4 hours[2]. Consistent with the previous results, rapamycin (AY-22989, MCE, USA) was administered at a concentration of 100 nM for 72 hours[3].

In vitro small interfering RNA (siRNA) or overexpression plasmid transfection

NRK-49F cells were seeded into 12-well culture dishes. After the cell confluence reached 50% on the following day, siRNA or overexpression plasmid was transfected into the cells using Lipofectamine 3000 (L3000150, Invitrogen Life Technologies, USA) in accordance with the manufacturer's instructions. After 6 hours, the cells were washed and cultured overnight in fresh DMEM containing 0% FBS, and then stimulated with 10 ng/mL of TGF-β1 for 72 hours. The siRNA sequence for rat *Kdm5b* interference was as follows: forward primer, 5'-GGAAACGAGAUGCAAUCAA(dT)(dT)-3'; reverse primer, 5'-UUGAUUGCAUCUCGUUUCC(dT)(dT)-3'. The siRNA sequence for rat

Tsc1 interference was as follows: forward primer, 5’-CCUGCAAGACCACAUGAUAA-3’; reverse primer, 5’-UUAUCACUGGGCUCCUAGGA-3’. The TSC1 overexpression plasmid was constructed by Obio Technology.

Western blotting analysis

Kidney tissues or cells were lysed in RIPA lysis buffer containing phenylmethylsulfonyl fluoride (PMSF). Immunoblotting experiments were conducted as described previously. The primary antibodies used were as follows: anti-USP7 (1:1000, 4833, Cell Signaling Technology, USA), anti-α-SMA (1:1000, A2547, Sigma, USA), anti-fibronectin (1:1000, ab2413, Abcam, USA), anti-glyceraldehyde-3-phosphate dehydrogenase (GAPDH, 1:10000, 60004-1-Ig, Proteintech, USA), anti-KDM5B (1:500, MA5-51950, ThermoFisher, USA), anti- mTOR (1:1000, A11355, Abclonal, China), anti-ubiquitin (1:500, sc-8017, Santa Cruz, USA), anti-phosphorylated mammalian target of rapamycin (p-mTOR, 1:1000, 67778-1-Ig, Proteintech, USA), anti-p70 ribosomal protein S6 kinase (p70 S6K, 1:1000, A4898, Abclonal, China), anti-ribosomal protein S6 (S6, 1:1000, 66886-1-Ig, Proteintech, USA), anti-phosphorylated p70 ribosomal protein S6 kinase (p-p70 S6K, 1:1000, AP0502, Abclonal, China), anti-phosphorylated ribosomal protein S6 (p-S6, 1:1000, AP1328, Abclonal, China), anti- TSC1 (1:1000, A0720, Abclonal, China), anti- H3K4me3 (1:1000, A22146,

Abclonal, China) , and anti-histone H3 (1:1000, 9715, Cell Signaling Technology, USA).Band intensity was quantitatively analyzed using ImageJ software.

RNA extraction and real-time quantitative reverse transcription polymerase chain reaction (qRT-PCR)

RNA extraction and real-time qRT-PCR were performed as described previously[4]. The mRNA levels were normalized according to GAPDH levels. The specific oligonucleotide primers were as follows: rat KDM5B forward primer, 5'-ACTGAGAGCAAAGAGACGGC-3', reverse primer, 5'-TTCGTCTCCACATCTTGGGC-3'; rat GAPDH forward primer, 5'-AGGTCGGTGTGAACGGATTTG-3', reverse primer, 5'-TGTAGACCATGTAGTTGAGGTCA-3'.

Immunoprecipitation (IP) and Co-immunoprecipitation (Co-IP)

Cells seeded into 10 cm culture dishes were collected in immunoprecipitation lysis buffer containing a protease inhibitor mixture and PMSF. Immunoprecipitation was performed using the antibodies anti-USP7 (1:25, 4833, Cell Signaling Technology, USA), anti-KDM5B (1:20, MA5-51950, ThermoFisher, USA), and anti-IgG (1:20, 3900, Cell Signaling Technology, USA) and protein A/G magnetic beads (1:10, 88802, Thermo Fisher Scientific,

USA) in accordance with the manufacturer's protocol. Captured antigen-antibody complexes were eluted with 2× SDS and analyzed by Western blotting assay. IgG served as a negative control.

Construction of overexpression virus and screening for stable transfected cells with knockout virus infection

Flag-USP7 lentivirus and CRISPR-Cas9 lentivirus targeting USP7 were constructed by Shanghai GeneChem Co., Ltd. The gene sequence used to generate single guide RNA targeting USP7 was 5’-CTATGTCGGCTTAAAGAATC-3’. NRK-49F cells were screened with puromycin after infection and verified by RT-PCR and Western blotting assays.

Chromatin immunoprecipitation (ChIP)-quantitative polymerase chain reaction (qPCR)

ChIP experiment was performed as described previously. Briefly, to prepare samples for ChIP, 1×10^{7} NRK-49F cells were taken from each sample, and crosslinked with 1% formaldehyde (without methanol) for 15 minutes. ChIP was then performed using a ChIP kit (RK20258, Abclonal). Chromatin was subsequently fragmented into approximately 200-bp fragments by sonication. Fragmented chromatin was incubated overnight with specific primary antibodies targeting KDM5B or H3K4me3 at 4 °C with gentle rotation. Protein

G magnetic microbeads were then added to the samples, followed by incubation under rotation for 2 hours. Subsequently, the microbeads were washed successively with washing buffers of varying salt concentrations and finally eluted with a buffer containing 1% SDS. The obtained antibody-chromatin complexes were sequentially subjected to reverse crosslinking and DNA purification steps. The resulting DNA samples were subjected to qRT-PCR using the following primers: P1 forward, 5’-TAAATCCCCAACCCCTCCTCTC-3’, reverse, 5’-CTCTCGGGTTGGATTTCACAT-3’; P2 forward, 5’-AACCCAAAGACCCACCCAAA-3’, reverse, 5’-ACCACCACTTGGCATTGCTT-3’; P3 forward, 5’-GTTGCTGTAGCTCGTTGCTT-3’, reverse primer, 5’-GCGGTGACAAGGTGTCAAGT-3’; P4 forward, 5’-GGGAAATAGGAACGCCCCAC-3’, reverse, 5’-ATCCAAACCAGGTCCCTCTG-3’. These primers were used to determine the binding of TSC1 and KDM5B, as well as the enrichment degree of H3K4me3 in NRK-49F cells. To determine the H3K4me3 modification level at the *Tsc1* promotor in mouse kidney tissues, the following primers were used: forward, 5’-GGAGCGTAGTAGAGACCCGA-3’; reverse, 5’-TCCCCCTCAGCTGTTTACCT-3’.

Reference

1. Zhang B, Li J, Wang Y, Liu X, Yang X, Liao Z, et al. Deubiquitinase USP7

stabilizes KDM5B and promotes tumor progression and cisplatin resistance in nasopharyngeal carcinoma through the ZBTB16/TOP2A axis. Cell Death Differ. 2024;31(3):309-321. https://doi.org/10.1038/s41418-024-01257-x.

2. Shi Y, Tao M, Chen H, Ma X, Wang Y, Hu Y, et al. Ubiquitin-specific protease 11 promotes partial epithelial-to-mesenchymal transition by deubiquitinating the epidermal growth factor receptor during kidney fibrosis. Kidney Int. 2023;103(3):544-564. https://doi.org/10.1016/j.kint.2022.11.027.

3. Nam H J, Han J H, Yu J, Cho C S, Kim D, Kim Y E, et al. Autophagy induction enhances homologous recombination-associated CRISPR-Cas9 gene editing. Nucleic Acids Res. 2025;53(7): https://doi.org/10.1093/nar/gkaf258.

4. Gu Y, Chen J, Zhang H, Shen Z, Liu H, Lv S, et al. Hydrogen sulfide attenuates renal fibrosis by inducing TET-dependent DNA demethylation on Klotho promoter. Faseb j. 2020;34(9):11474-11487. https://doi.org/10.1096/fj.201902957RR.

Supplementary Table S1. Clinical characteristics of the participants enrolled in this study

CKD 1: chronic kidney disease stage 1, CKD 2: chronic kidney disease stage 2, CKD 3: chronic kidney disease stage 3, CKD 4: chronic kidney disease stage 4, CKD 5: chronic kidney disease stage 5, SBP: systolic blood pressure, DBP: diastolic blood pressure, Hb: hemoglobin, FPG: fasting plasma glucose, TG: triglyceride, TC: total cholesterol, SCr: serum creatinine, SUA: serum uric acid, BUN: blood urea nitrogen.

	CKD 1 (n=9)	CKD 2 (n=9)	CKD 3 (n=9)	CKD 4 (n=9)	CKD 5 (n=9)	*P*
Sex (male)	4 (44.4%)	6 (66.7%)	5 (55.6%)	4 (44.4%)	5 (55.6%)	0.870
Age (years)	54.0±14.8	51.6±13.0	46.1±17.3	49.7±14.6	55.6±12.3	0.662
SBP (mmHg)	131.4±21.1	137.7±18.0	135.8±11.7	132.2±16.5	142.1±12.8	0.644
DBP (mmHg)	78.3±9.0	75.7±8.6	81.4±14.7	84.2±9.2	82.7±8.3	0.670
Hb (g/L)	128.4±22.0	116.1±17.7	106.1±13.5	102.3±18.4	83.9±14.9	<0.001
FPG (mmol/L)	6.5±1.6	6.1±1.9	6.0±0.4	5.7±1.4	5.4±1.9	0.986
HbA1c (%)	5.3±1.3	6.7±1.2	5.7±0.75	5.8±1.2	6.1±1.4	0.149
Serum albumin (g/L)	28.8±8.8	29.8±8.2	36.4±5.9	32.9±8.9	35.8±5.2	0.143
TG (mmol/L)	1.8±0.6	2.6±2.3	2.2±1.0	1.9±0.8	2.0±1.1	0.718
TC (mmol/L)	7.2±2.6	7.4±1.7	6.5±0.9	5.9±0.9	6.6±2.3	0.436
SCr (μmol/L)	102.1±36.3	116.2±49.8	165.3±77.4	287.3±97.7	454.6±189.9	<0.001
SUA (μmol/L)	344.3±78.8	370.2±94.4	368.8±77.2	344.1±101.2	427.1±109.7	0.331
BUN (mmol/L)	6±2.7	8.1±2.3	8.5±2.1	7.7±1.7	16.4±4.6	<0.001

Supplementary Table S2. The amino acid sites and distances of the interaction between protein USP7 and protein KDM5B

	**USP7**	**KDM5B**	Distance (Å)
**hydrogen bond**	ASN1069	GLU1025	3.0
	GLN1066	SER1055	2.5
	HIS1048	ARG1241	3.3
	ILE1042	ARG1058	3.5
	GLY1046	ARG1243	2.5
	GLN1049	ARG1058	3.3
**salt bridge**	HIS1072	GLU1025	3.9
	LYS 335	GLU1230	2.8

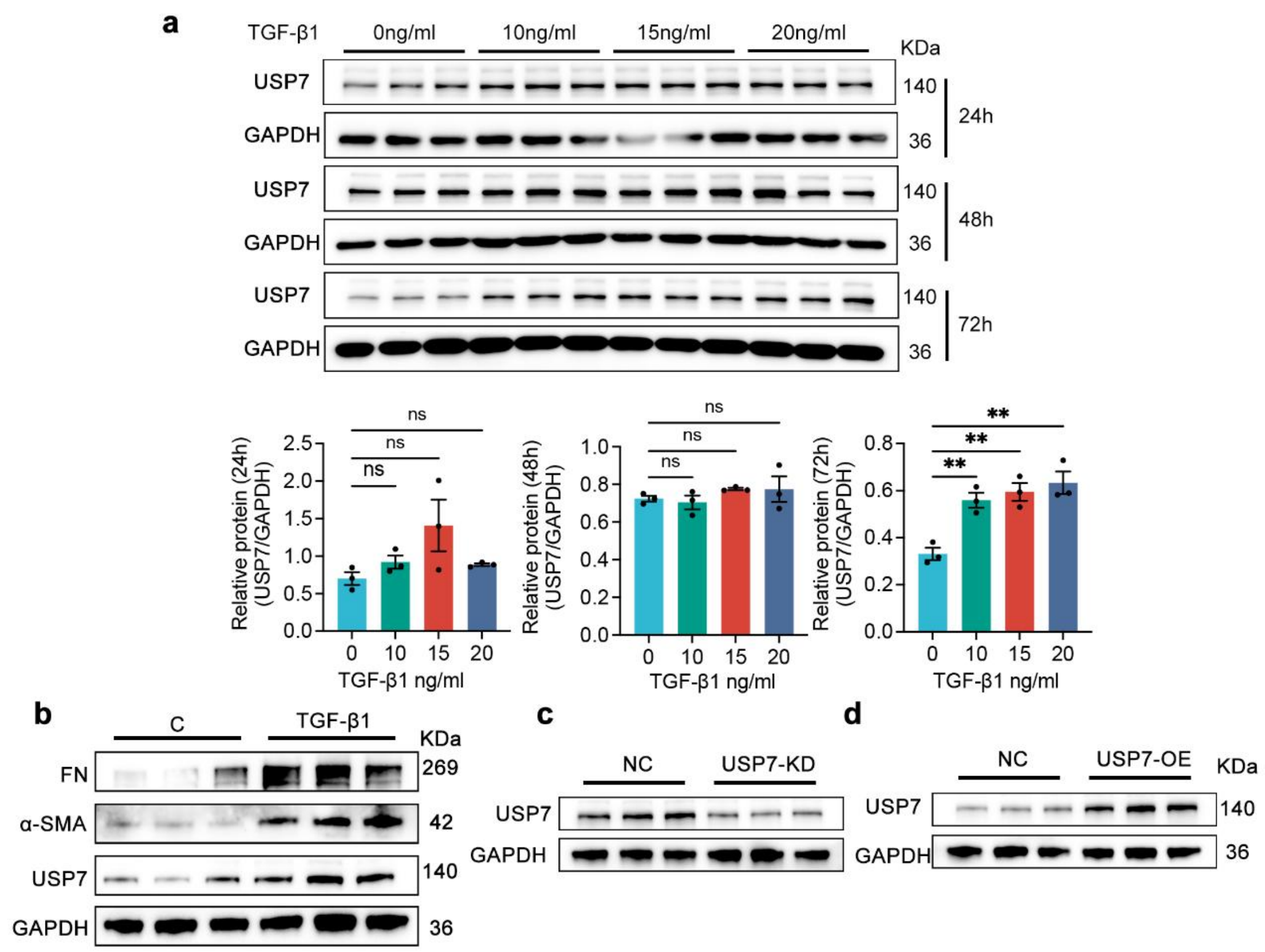

Supplementary Figure S1. TGF-β1 induced the upregulation of USP7 in NRK-49F Cells

(a) NRK-49F cells were treated with different concentrations of TGF-β1, and the cells were collected at different time points for protein immunoblot analysis. Statistical charts were shown in the lower panel. (b) NRK-49F were incubated with or without TGF-β1(10 ng/ml) for 72 hours, and then the cells were collected for immunoblotting analysis. (c and d) NRK-49F cells were infected with constructed viruses (USP7 knockdown virus-USP7-KD, USP7 overexpression virus-USP7-OE, negative control virus-NC) to construct virus stably transfected mixed clonal strains, and the cells were collected for immunoblotting analysis.. All data are presented as mean ± SEM. n = 3. “ns” indicates no significant

difference, **$P < 0.01$.

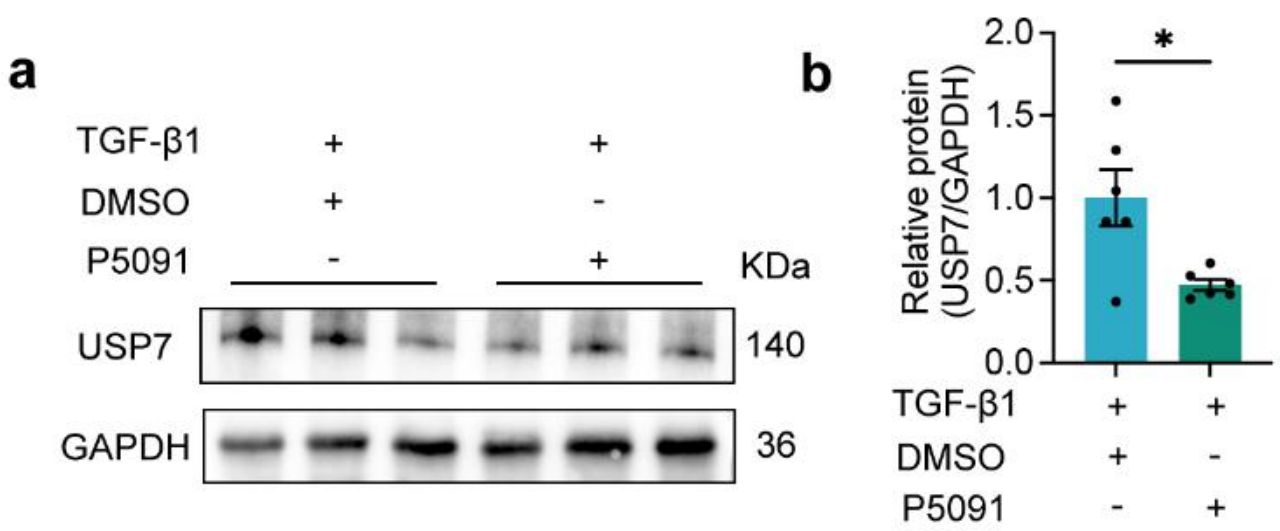

Supplementary Figure S2. Effect of USP7 inhibitor P5091 on USP7 protein expression

(a) NRK-49F cells were incubated with or without the USP7 inhibitor P5091 (10 μM) under the stimulation of TGF-β1 (10 ng/ml) for 72 hours, and then the cells were collected for immunoblotting analysis. (b) Statistical charts for figure a. All data are presented as mean ± SEM. n = 3. *$P < 0.05$.

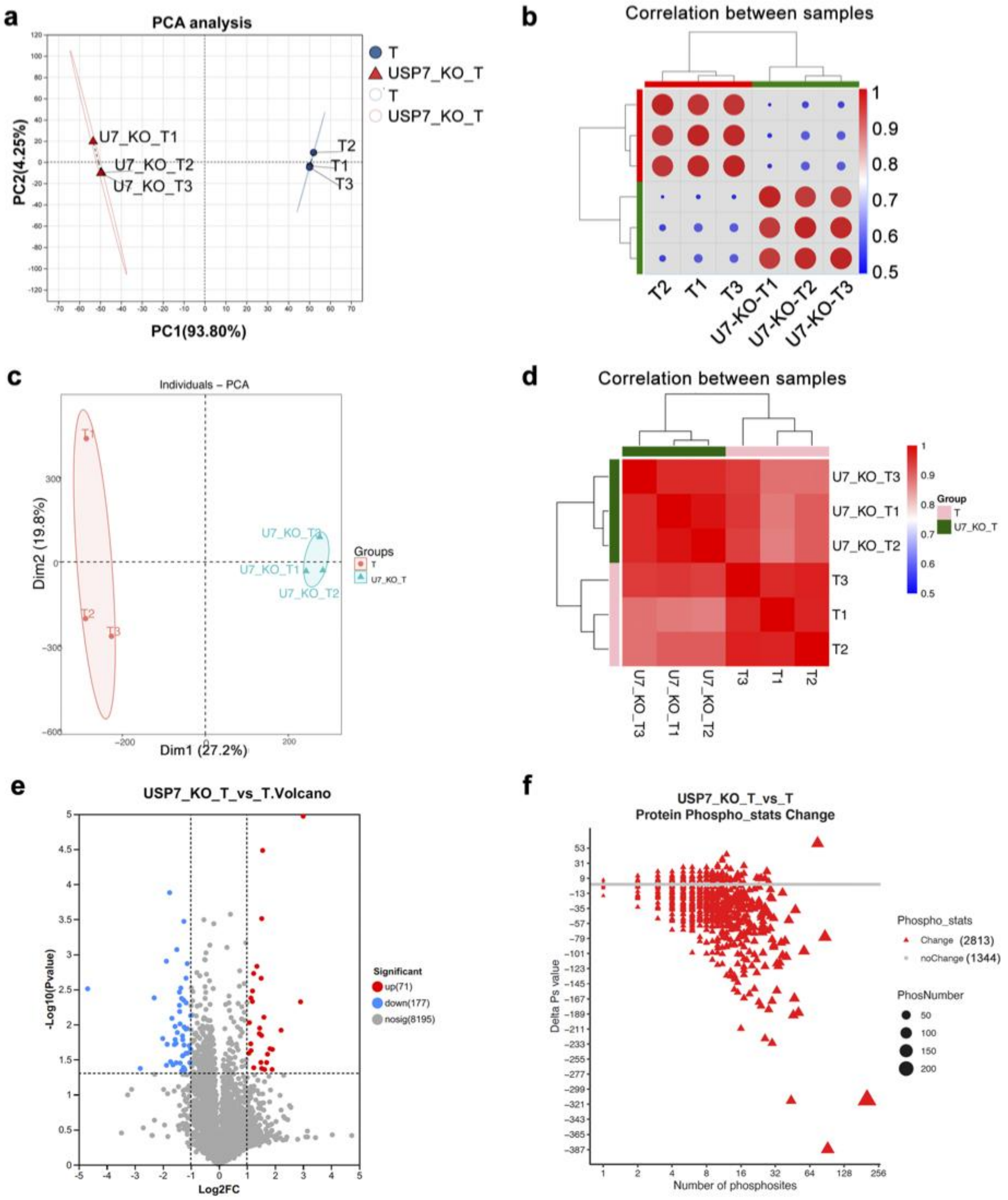

Supplementary Figure S3. Proteomic sequencing and phosphoproteomics analyses between USP7-KD and NC stably transfected NRK-49F cells stimulated by TGF-β1

(a) Principal component analysis (PCA) of proteomes in NC and USP7-KD stably transfected NRK-49F cells treated with TGF-β1. (b) Correlation of

proteome profiles between NC cells and USP7-KD cells treated with TGF-β1. (c) PCA of phosphoproteomes in NC and USP7-KD stably transfected NRK-49F cells treated with TGF-β1. (d) Correlation of phosphoproteomics profiles between NC cells and USP7-KD cells treated with TGF-β1. (e)The volcano plot showed the differentially expressed proteins in the proteomic sequencing of USP7-KD and NC stably transfected NRK-49F cells stimulated by TGF-β1. (f) The scatter plot showed the differentially phosphorylated proteins in the phosphoproteomics of USP7-KD and NC stably transfected NRK-49F cells stimulated by TGF-β1. n = 3.

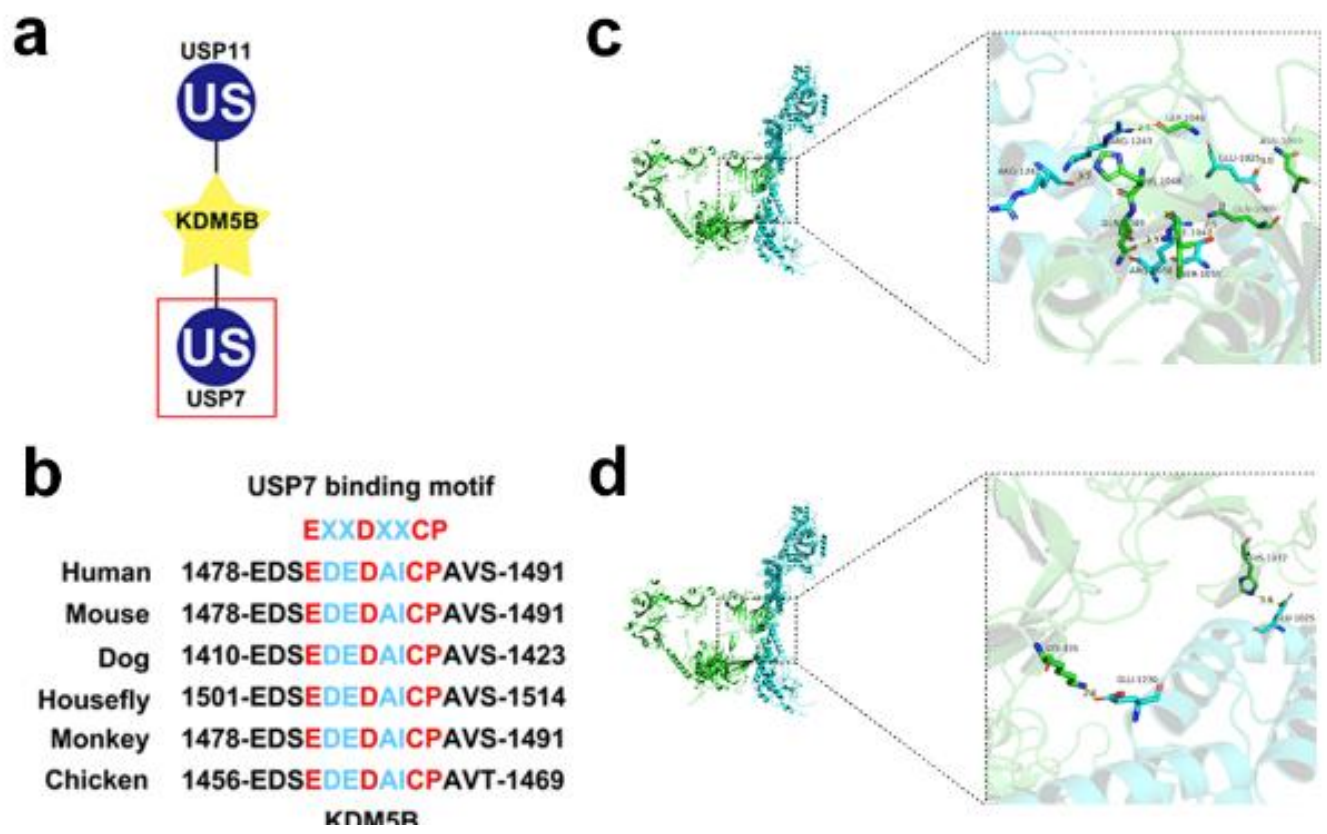

Supplementary Figure S4. Prediction of interaction between USP7 and TSC1

(a) UbiBrowser (ubiquitination browser) shows the deubiquitinating enzymes (DUBs) that may interact with KDM5B. (b) A schematic diagram depicts the deubiquitination consensus motif of USP7 for KDM5B. (c and d) Protein-protein interaction diagrams and the names of amino acid residues involved in

hydrogen bond (c) and salt bridge (d) interactions between proteins, where the cyan part represents the KDM5B protein and the green part represents the USP7 protein.

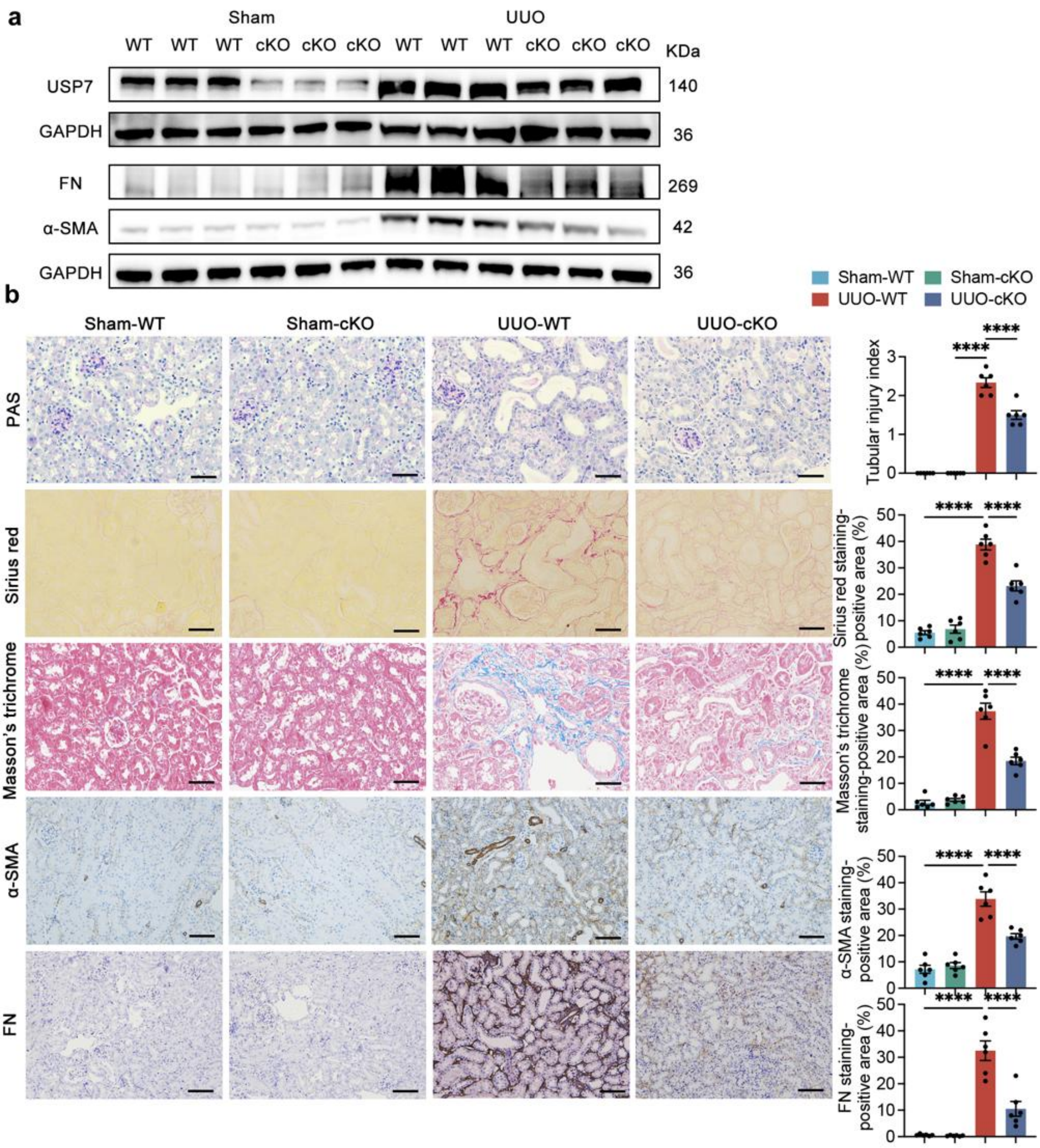

Supplementary Figure S5. Fibroblast-specific depletion of USP7 alleviates renal fibrosis induced by UUO

(a) Immunoblotting analysis was performed on the whole kidney tissue lysates

of cKO mice and WT mice receiving sham or UUO operation. (b) Microscopic photographs show the PAS staining (Bars=50 μm), Masson's trichrome staining (Bars=50 μm), Sirius red staining (Bars=50 μm), and immunohistochemical staining (Bars = 100 μm) of α-SMA and FN of the kidneys. The morphological changes of renal tubular injury were scored according to the PAS staining as described in the Methods section. The other four pictures show the positive areas of Masson's trichrome (blue), Sirius red (red), and immunohistochemical staining of α-SMA and FN. All data are presented as mean ± SEM. n = 6. ****$P < 0.0001$.

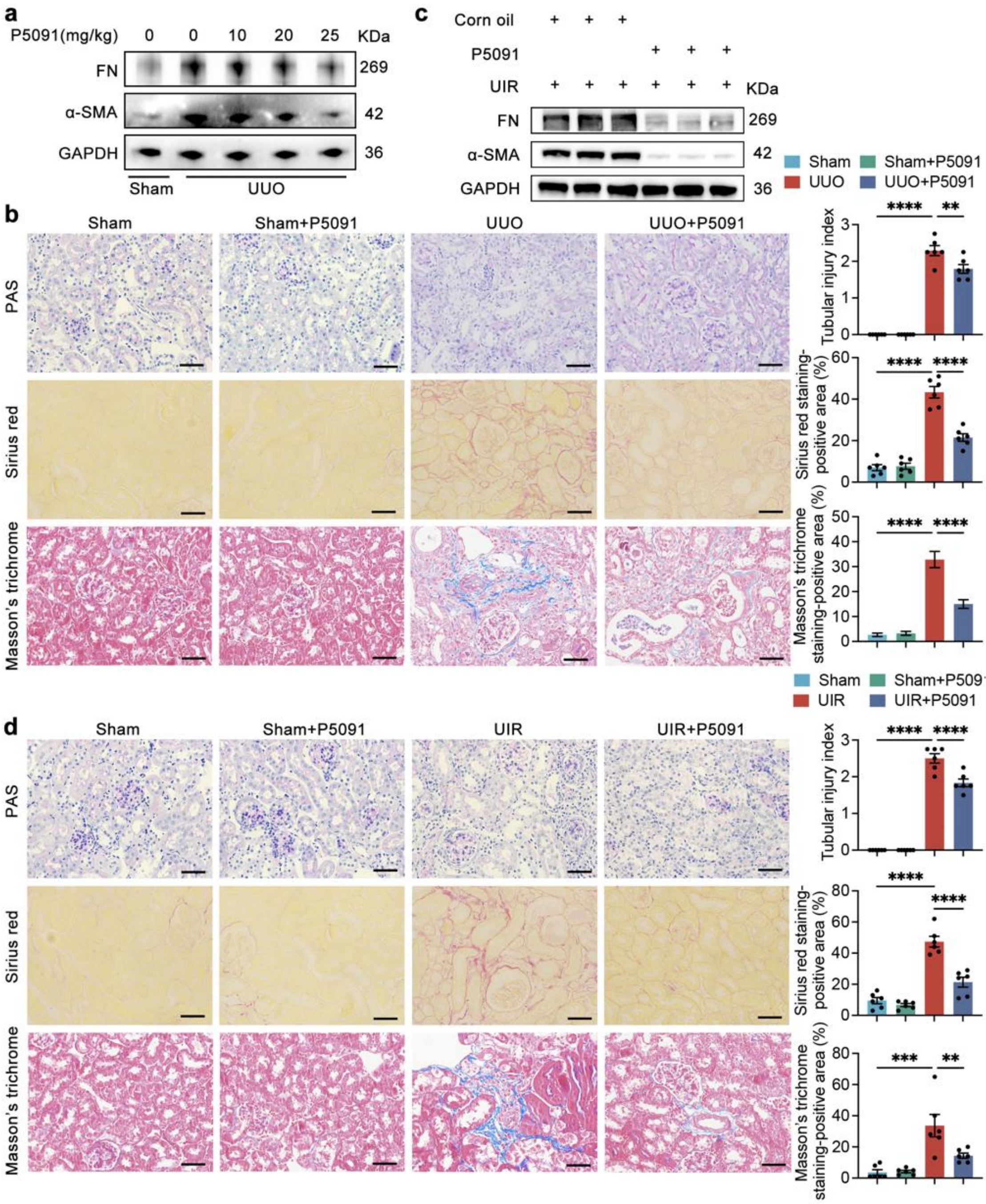

Supplementary Figure S6. The inhibitor of USP7 (P5091) alleviates renal fibrosis induced by UIR and UUO

(a) Wild-type mice received different doses of P5091 (as dedicated, i.p.) following sham or UUO operation. Renal protein expression of α-SMA and FN

were detected. Administration of P5091 at 25 mg/kg resulted in the lowest expression of α-SMA and FN in the kidneys of UUO mice. (b) PAS staining, Sirius red staining and Masson's trichrome staining of the kidneys in Sham or UUO mice receiving P5091 administration (25 mg/kg). Bars=50 μm. (c) Wild-type mice received 25 mg/kg P5091 (i.p.) following sham or UIR operation. Renal protein expression of and FN were detected. (d) PAS staining, Sirius red staining and Masson's trichrome staining of the kidneys in Sham or UIR mice receiving P5091 administration (25 mg/kg). Bars=50 μm. All data are presented as mean ± SEM. n = 6. ***P* < 0.01, ****P* < 0.001, *****P* < 0.0001.

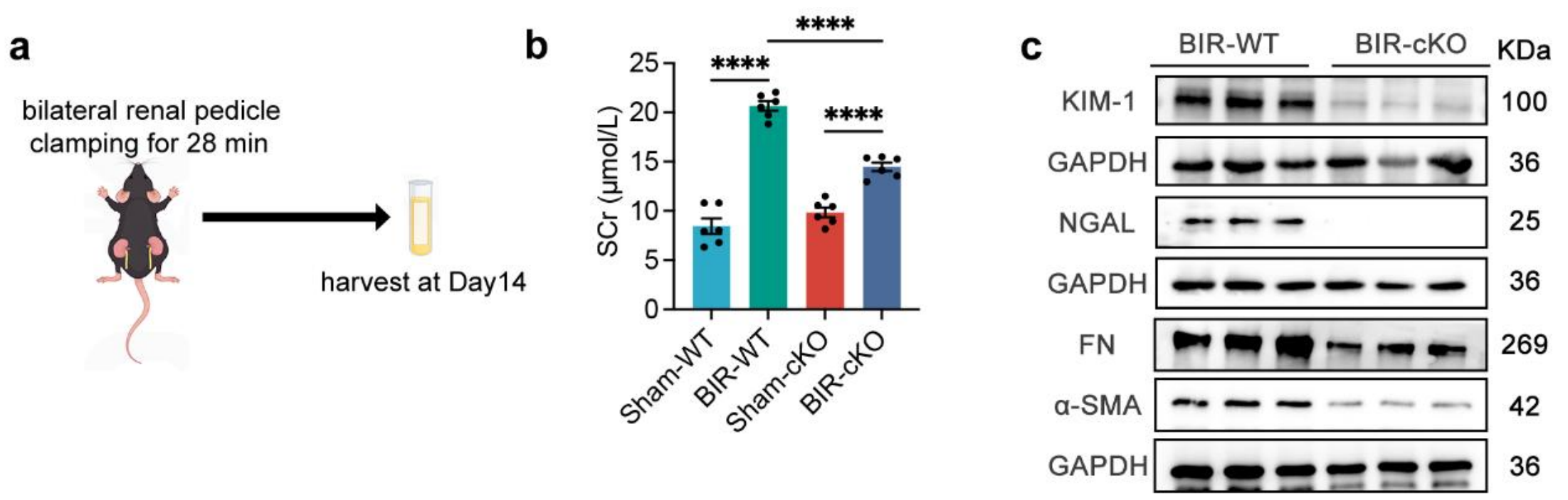

Supplementary Figure S7. Fibroblast-specific depletion of USP7 attenuates renal function dysfuction induced by BIR

(a) Flowchart of the mouse BIR experiment. (b) SCr were detected in cKO mice and WT mice receiving sham or BIR operation. (c) Renal protein expression of KIM-1, NGAL, α-SMA and FN were detected by Western blotting. All data are presented as mean ± SEM. n = 6. ***P* < 0.01, *****P* < 0.0001.

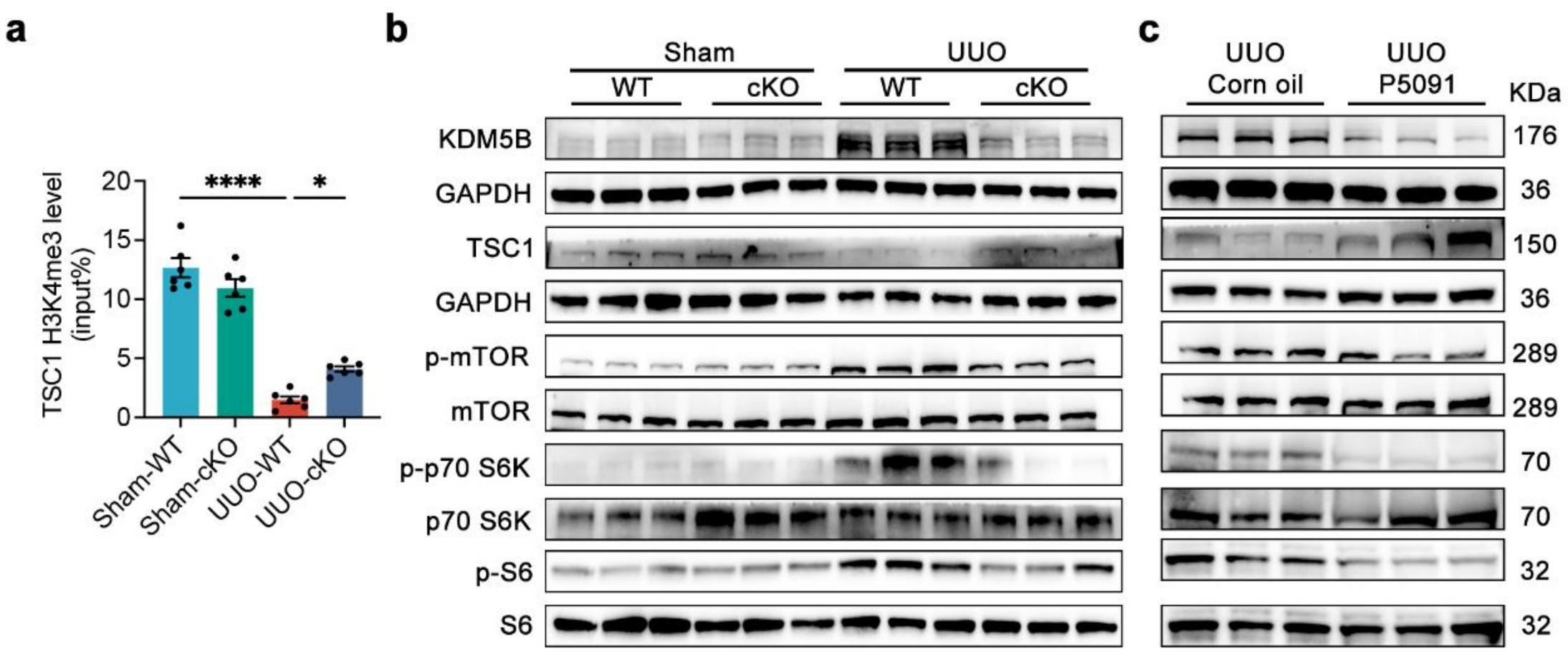

Supplementary Figure S8. USP7 conditional knockout and P5091 intervention inhibit KDM5B expression, restore TSC1 protein level, and inhibit mTOR signaling pathway activation in UIR mouse models

(a) H3K4me3 levels at *Tsc1* promotor in each group. (b) Renal protein expression in cKO mice and WT mice receiving sham or UUO operation. (c) Renal protein expression in WT mice receiving P5091 administration after UUO operation. All data are presented as mean ± standard deviation. n = 6. $*P < 0.05$, $****P < 0.0001$.
